# Ionizing radiation exposure of stem cell-derived chondrocytes affects their gene and microRNA expression profiles and cytokine production

**DOI:** 10.1038/s41598-021-86230-1

**Published:** 2021-04-05

**Authors:** Ewelina Stelcer, Katarzyna Kulcenty, Marcin Rucinski, Marta Kruszyna-Mochalska, Agnieszka Skrobala, Agnieszka Sobecka, Karol Jopek, Wiktoria Maria Suchorska

**Affiliations:** 1 Department of Electroradiology, Poznan University of Medical Sciences, Garbary 15th, 61-866 Poznan, Poland; 2 Radiobiology Lab, Greater Poland Cancer Centre, Garbary 15th Street, 61-866 Poznan, Poland; 3 Department of Histology and Embryology, Poznan University of Medical Sciences, Swiecickiego 6 Street, 60-781 Poznan, Poland; 4 Department of Medical Physics, Greater Poland Cancer Centre, Garbary 15th, 61-866 Poznan, Poland; 5 Department of Head and Neck Surgery, Poznan University of Medical Sciences, Garbary 15th, 61-866 Poznan, Poland

**Keywords:** Induced pluripotent stem cells, Biological physics, Molecular medicine

## Abstract

Human induced pluripotent stem cells (hiPSCs) can be differentiated into chondrocyte-like cells. However, implantation of these cells is not without risk given that those transplanted cells may one day undergo ionizing radiation (IR) in patients who develop cancer. We aimed to evaluate the effect of IR on chondrocyte-like cells differentiated from hiPSCs by determining their gene and microRNA expression profile and proteomic analysis. Chondrocyte-like cells differentiated from hiPSCs were placed in a purpose-designed phantom to model laryngeal cancer and irradiated with 1, 2, or 3 Gy. High-throughput analyses were performed to determine the gene and microRNA expression profile based on microarrays. The composition of the medium was also analyzed. The following essential biological processes were activated in these hiPSC-derived chondrocytes after IR: "apoptotic process", "cellular response to DNA damage stimulus", and "regulation of programmed cell death". These findings show the microRNAs that are primarily responsible for controlling the genes of the biological processes described above. We also detected changes in the secretion level of specific cytokines. This study demonstrates that IR activates DNA damage response mechanisms in differentiated cells and that the level of activation is a function of the radiation dose.

## Introduction

Upper airway reconstruction, including tracheal and laryngotracheal resection and reconstruction, is a common surgical procedure after resection of tumours or stenotic inflammatory lesions^1^. However, restoring proper airway function in these cases is difficult^2^. In recent years, the use of exogenous autologous stem or progenitor cells, which include autologous adipose-derived stem cells, mesenchymal stromal cells, and induced pluripotent stem cells (iPSC), has been proposed to improve airway reconstruction^3^. While the individual use of chondrocytes or mesenchymal stem cells (MSC) is, at present, not considered ideal for cell-based cartilage repair in the head and neck (H&N) area^4^, the combination of these two cell sources holds great promise for cartilage tissue engineering, as it reduces the required number of chondrocytes and attenuates most of the disadvantages of the individual cell types. Mixed cell cultures of chondrocytes and MSCs have also been shown to improve chondrogenesis and to reduce hypertrophy and tissue mineralization^5^.

Human iPSCs (hiPSCs) possess the ability to differentiate into chondrocytes to produce a cartilaginous matrix, thus obviating the need to biopsy healthy cartilage^6^. The most significant benefit of hiPSC-derived progenitor cells for cartilage regeneration is that they may provide an unlimited supply of chondrogenic cells for in vivo applications. Consequently, cell-based therapy has been recognized as a potentially effective approach to healing tracheal cartilage^7^. Imaizumi et al.^8^ suggested that hiPSCs could be a new cell source to regenerate tracheal cartilage. However, this strategy is not without risk given that those transplanted cells may eventually be subjected to ionizing radiation (IR) in patients who develop cancer^9^. At present, published data on irradiated hiPSC-derived chondrocytes are limited. Previously, our group (Stelcer et al.^10^) showed that the differentiation process has a major impact on the DNA damage response (DDR) mechanisms of irradiated cells^10^. Nevertheless, more data are needed to better elucidate the key aspects of DDR mechanisms activated in hiPSC-derived chondrocytes exposed to IR.

In this context, the main aim of the present study was to determine the global gene and microRNA expression profile of chondrocyte-like cells differentiated from hiPSCs placed in an Alderson phantom and irradiated according to a conventional radiotherapy regimen (1,2, or 3 Gy) in a laryngeal cancer model^11,12^.

Our findings revealed activation of the following key biological processes in hiPSC-derived chondrocytes after irradiation: “apoptotic process”, “cellular response to DNA damage stimulus”, and "regulation of programmed cell death". We also observed decreased expression of genes involved in the cell cycle and in cell division, and reduced expression of other genes. We identified the presence of various microRNAs responsible for controlling the genes of these three biological processes (e.g., hsa-miR-34a-5p, hsa-miR-551b-5p, hsa-miR-362-3p and hsa-miR-3187-3p, among others). Finally, we demonstrated significant changes in the secretion of several cytokines, including G-CSF, GRO-alpha, IP-10, and TNF-alpha after IR.

The findings of this study demonstrate that IR activates DDR mechanisms in differentiated cells and that the level of activation is a function of the radiation protocol applied, with activation increasing as a function of the radiation dose.

## Results

### Differentiated cells possess features of chondrocytes

**Figure 1:**
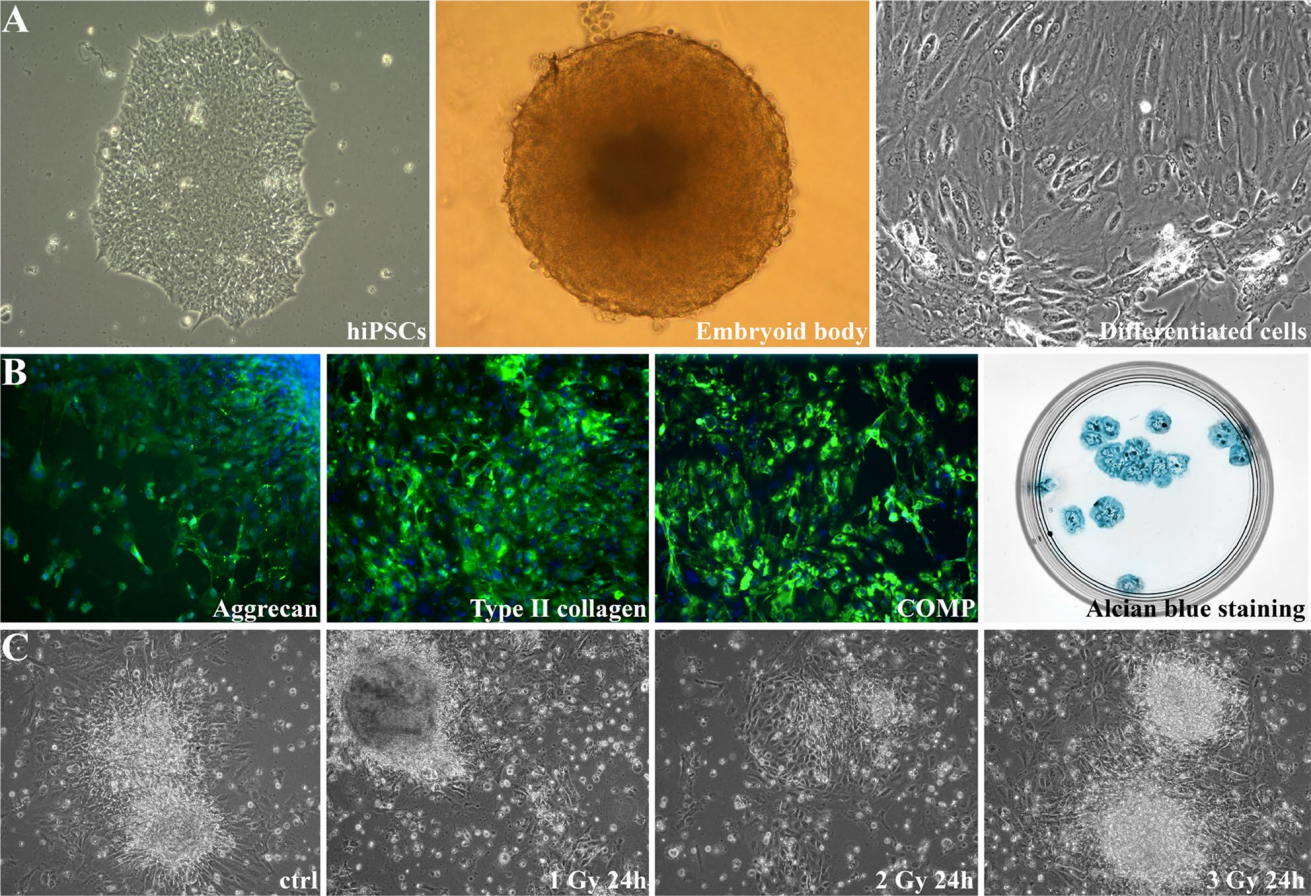
Human-induced pluripotent stem cells (hiPSCs) underwent chondrogenic differentiation in a medium supplemented with TGF-β3 via EBs (**A**). The obtained chondrocyte-like cells revealed markers characteristic of chondrocytes, including aggrecan, type II collagen, cartilage-oligomeric matrix protein (COMP), and proteoglycans visualized by alcian blue staining (**B**). Those cells were treated with ionizing radiation at 1, 2 and 3 Gy (**C**).

**Figure 2:**
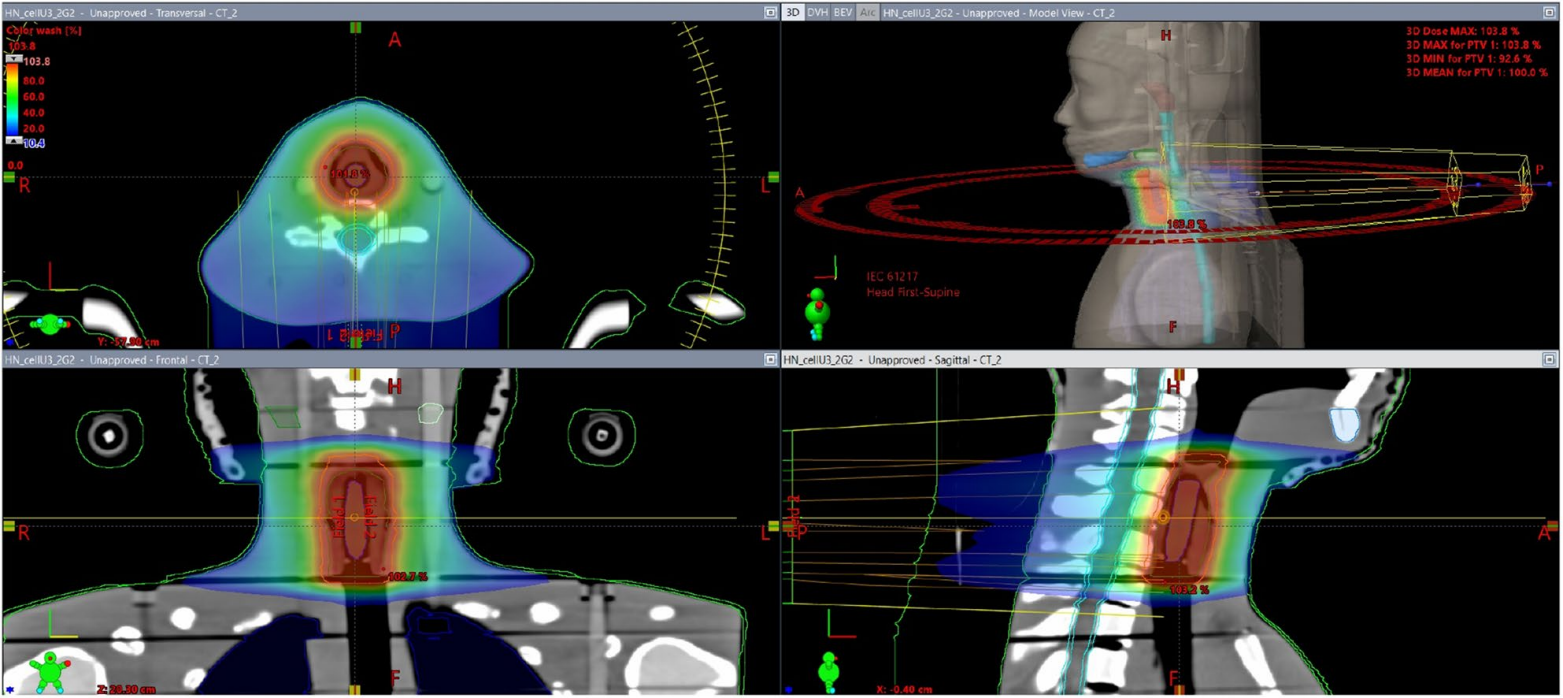
Dose distribution on axial, sagittal, and coronal views and a 3D view of VMAT plans created to irradiate the chondrocyte-like cells in the ART phantom. The air space simulating the esophagus was filled with wax surrounding an Eppendorf tube, as quasi-GTV (blue color), quasi-CTV (green color), and quasi PTV (orange color).

We first formed EBs from hiPSCs in a culture medium deprived of FGF-2 for 7 days. Those EBs were differentiated towards chondrocytes in the presence of TGF-β3 for 21 days (Fig. 1A). These chondrocyte-like cells exhibited the characteristics of chondrocytes, including expression of desirable markers such as COMP, type II collagen, and aggrecan (Fig. 1B and Supplementary Fig. 4). Glycosaminoglycan (GAG) deposition was marked by alcian blue staining. Blue staining and condensed cartilage-like nodules were evident in EBs formed during chondrogenic differentiation (Fig. 1B). After differentiation, the chondrocyte-like cells were irradiated according to conventional therapy protocols (1, 2, 3 Gy) (Fig. 2). After IR, no notable changes in cell morphology were observed (Fig. 1C); however, we clearly proved that IR treatment results in inhibition of cell growth (Supplementary Fig. 5), causes DSBs (Supplementary Fig. 6A,B) and leads to necrosis (Supplementary Fig. 7A,B).

### Microarray gene expression profiling: signaling pathways involving DNA repair, apoptosis, and the p53 cell cycle are significantly regulated in irradiated chondrocyte-like cells differentiated from hiPSCs

Affymetrix Human Gene 2.1 ST array strips were used to analyze whole gene expression and to perform a complete comparison of differentiated hiPSCs treated with IR (40,716 different transcripts). The general profiles of the whole gene expression in the cells at 1 h and 24 h post-treatment are presented in the volcano plots (Supplementary Fig. 1), where each blue dot represents one differentially expressed transcript.

We assumed the following selection criteria for changed gene expression: an expression fold difference > abs. 1.5 and an adjusted *p* value ≤ 0.05 with 20% false discovery rate (FDR) correction. According to these criteria, the number of downregulated or upregulated genes in the cells 1 h after IR (1, 2 and 3 Gy, respectively) versus non-irradiated controls was as follows: downregulated: 10, 0, 1 genes; upregulated: 6, 0, 1 genes. At 24 h, the numbers of significantly down- or upregulated genes were as follows: downregulated: 28, 40, and 70, respectively and upregulated: 7, 40 and 24, respectively (Supplementary Fig. 1). Given that changes at 1 h post-IR were barely perceptible, we decided to analyze only the material from cells at 24 h after irradiation.

**Figure 3:**
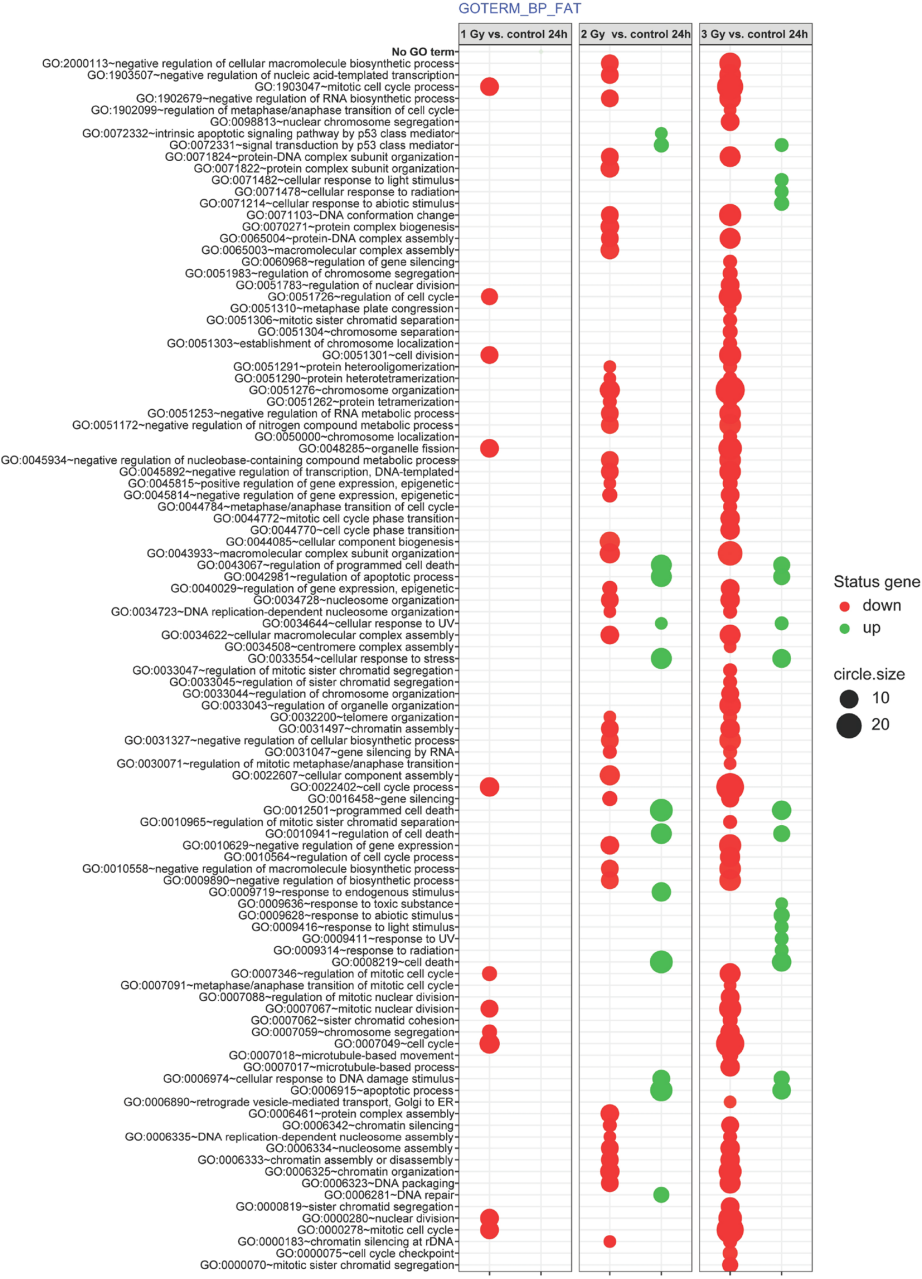
Bubble plot of differentially-expressed genes from each group (1 Gy vs. controls; 2 Gy vs. controls; 3 Gy vs. controls) assigned to gene ontology (GO) terms where the red color represents GO terms whose genes are downregulated while green corresponds to GO terms of upregulated genes.

Differentially expressed genes from each group (1 Gy vs. control; 2 Gy vs. control; 3 Gy vs. control) were then assigned to Gene Ontology (GO) (Fig. 3). The GO analysis showed that irradiation of hiPSC-derived chondrocytes significantly alters the expression of certain genes that play an essential role in the regulation of signaling pathways, *inter alia*: “regulation of programmed cell death” (0043067), “apoptotic process” (0006915), “DNA repair “(0006281), “signal transduction by p53 class mediator” (0072331), “cell cycle” (0007049), “cell division” (0051301), and “mitotic cell cycle” (0000278). Note that the hypofractionated dose (3 Gy) resulted in the greatest number of altered biological processes.

**Figure 4:**
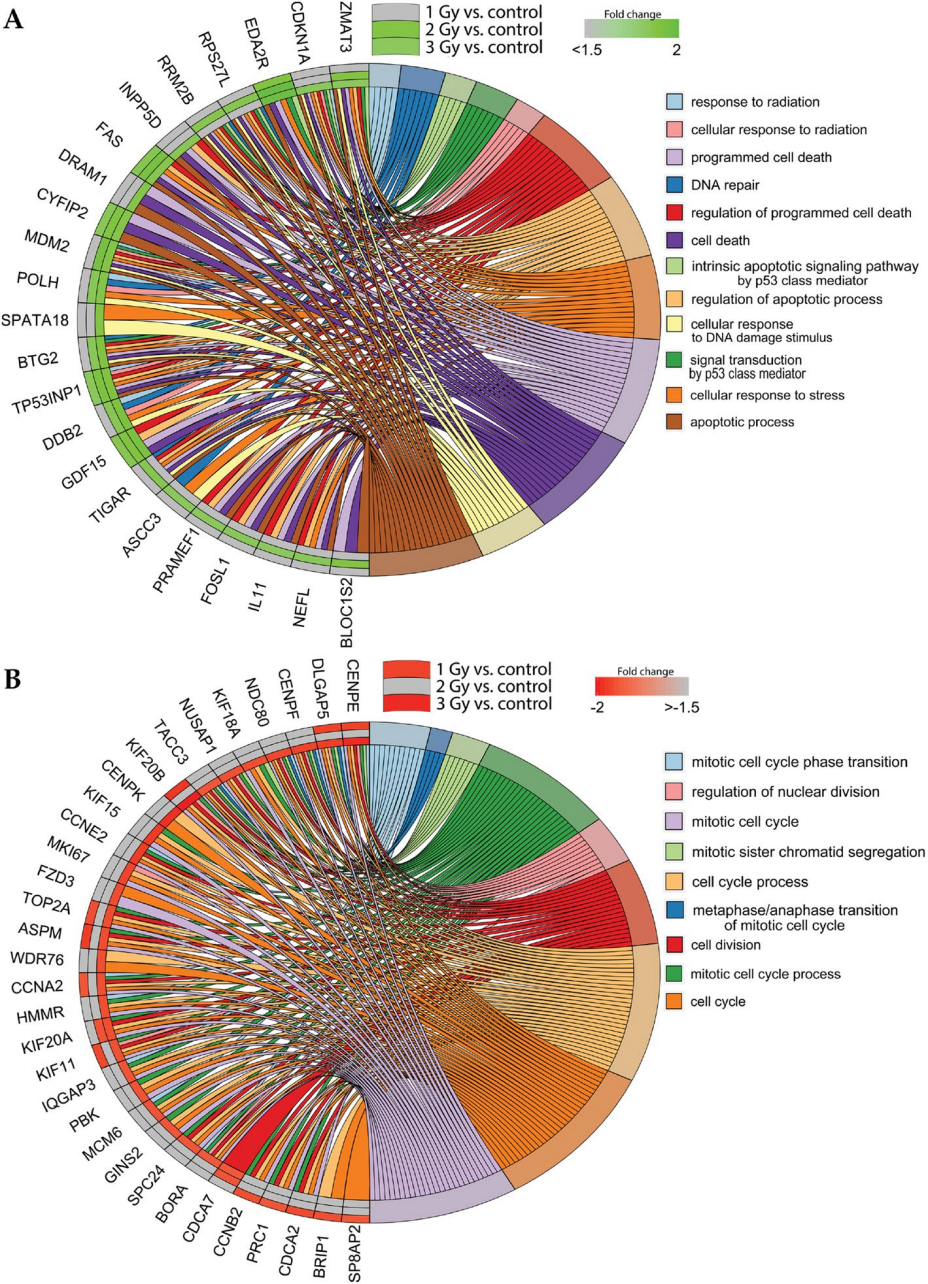
The relationship between particular genes belonging to GO terms mapped as circos plots. LogFC values and gene symbols are shown on the left side of the graph. All of the genes were upregulated (**A**) or downregulated (**B**) in at least one group in the irradiated cells (1, 2 and 3 Gy) compared to controls.

**Figure 5:**
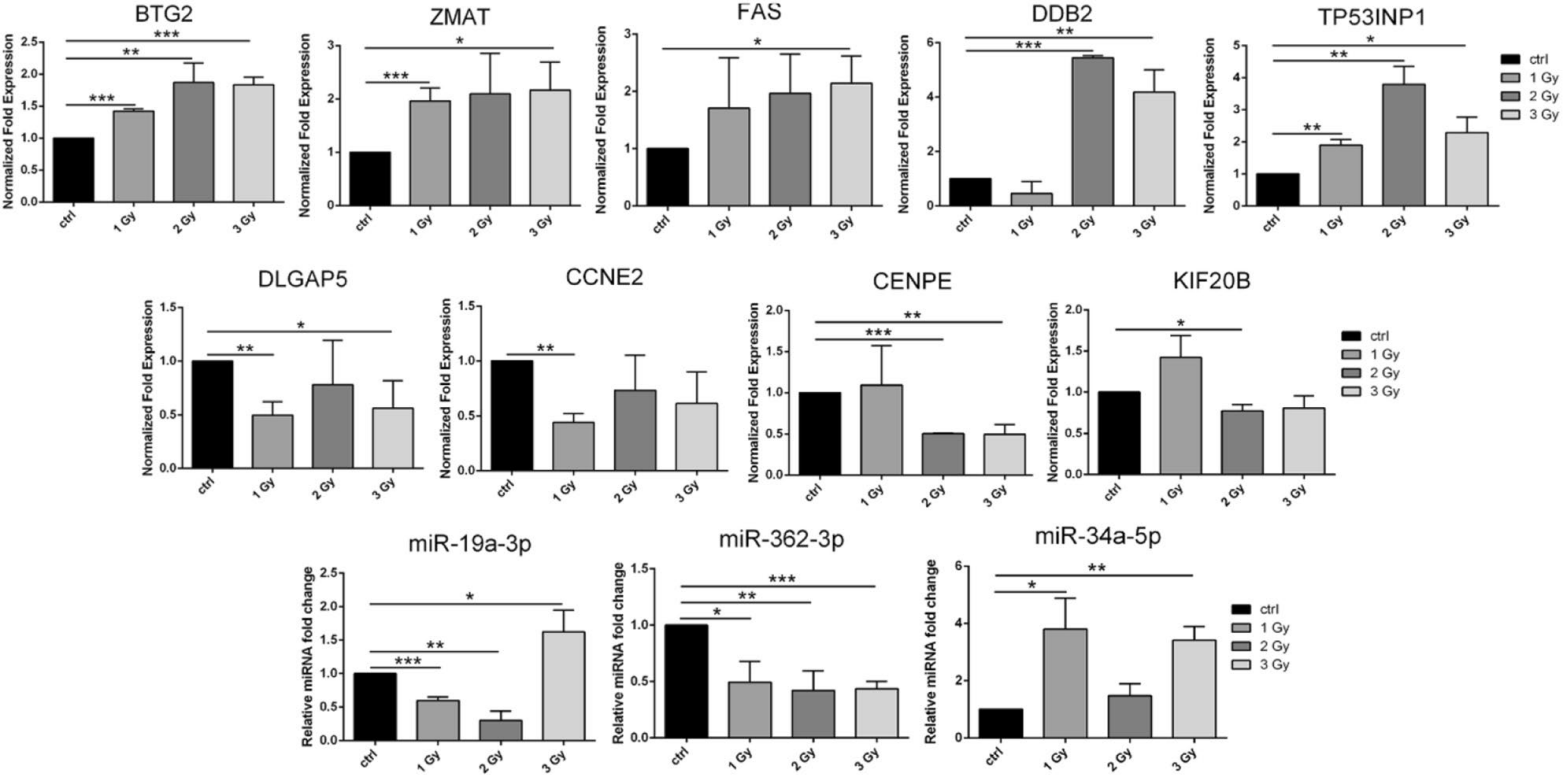
Expression of the most upregulated and downregulated mRNAs were: *BTG2*, *ZMAT*, *FAS*, *DDB2*, *TP53INP1*, *DLGAP5*, *CCNE2*, *CENPE*, *KIF20B* and microRNAs: hsa-miR-19a-3p, hsa-miR-362-3p, hsa-miR-34a-5p were validated by RT-qPCR.

Due to the structure of the GO database, single genes can often be assigned to many ontological terms. For this reason, the relationship between genes and GO terms were mapped with circos plots, with visualization of logFC values and gene symbols. All of those genes were either upregulated (Fig. 4A) or downregulated (Fig. 4B) in the irradiated cells compared to controls. The most upregulated or downregulated genes (*CCNE2*, *DLGAP5*, *KIF20B*, *FAS*, *TP53INP1*, *BTG2*, *DDB2*, *ZMAT3* and *CENPE*) were then subjected to further qPCR evaluation (Fig. 5). All these genes are involved in several different pathways activated after IR, including reprogrammed cell death, response to radiation, and mitotic cell cycle processes.

The expression of genes engaged in DDR in the irradiated hiPSC-derived chondrocytes was high when compared to non-irradiated cells. Genes involved in apoptosis were also upregulated in the irradiated chondrocyte-like cells. By contrast, expression of genes involved in cell division was strongly downregulated in irradiated cells.

**Figure 6:**
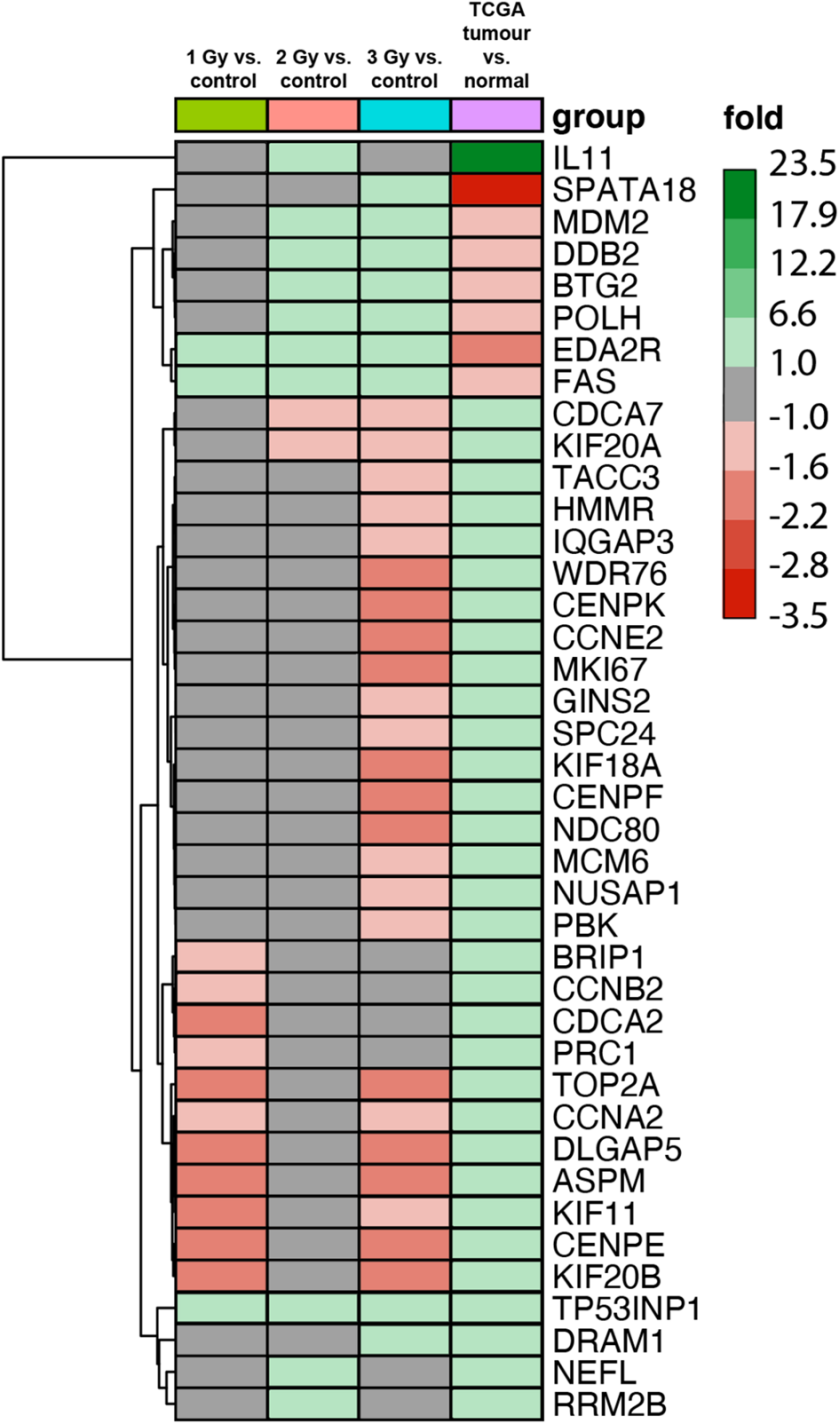
Heatmap with hierarchic clusterization of differentially expressed genes involved in the control of "cell cycle" and "cell division". The values represent the average fold changes after IR (1, 2, 3 Gy) versus controls. For TCGA data, the values show fold change from laryngeal tumor samples versus normal samples.

We compared expression of the most downregulated genes engaged in DDR in the hiPSC-derived chondrocytes (1 Gy vs. control; 2 Gy vs. control; 3 Gy vs. control) to those obtained from laryngeal tumor tissues compared to normal samples (data from the Cancer Genome Atlas [TCGA] were obtained through the FireBrowse server (http://gdac.broadinstitute.org/)^13^ (Fig. 6). After IR, the expression of genes engaged in the cell cycle and cell division (e.g., *CENPE*, *KIF11*, *NUSAP1* and *MCM6)* was strongly downregulated in hiPSC-derived cells. By contrast, the gene expression profile of tumor samples were strongly upregulated compared to normal samples.

In addition, we observed a radiation dose-dependent upregulation of some genes (e.g. *SPATA18*, *DDB2* and *EDA2R*) involved in DDR in the differentiated cells. By contrast, the comparison between laryngeal tumor samples and normal samples did not find any upregulation of expression of genes involved in DDR. The differing gene expression profile between (irradiated vs. non-irradiated) chondrocyte-like cells and (tumor vs. normal) laryngeal samples may indicate some degree of safety (i.e., low tumorigenic potential) of using differentiated cells that may be subjected to IR.

### Microarray microRNA expression profiling: irradiated chondrocyte-like cells are characterized by distinct miRNA expression profile compared to non-irradiated cells

A GeneChip™ miRNA 4.1 Array Strip was used to analyze and compare the miRNA expression profiles of chondrocytes derived from hiPSCs (controls) to irradiated chondrocyte-like cells. The miRNA expression profile of the non-irradiated and irradiated cells differed notably. The cut-off criteria used were as follows: fold difference > abs. 1.5 and adjusted *p* value ≤ 0.05, with 20% FDR correction. The number of downregulated or upregulated miRNAs was as follows: downregulated: 13, 31, 29 miRNAs (1, 2 and 3 Gy, respectively) and upregulated: 13, 25, 3 miRNAs (1, 2 and 3 Gy, respectively) that met cut-off criteria in non-irradiated differentiated cells versus hiPSC cells; those results are shown as a volcano plots (Supplementary Fig. 2). The smallest change in microRNA expression was observed in cells that received the hyperfractionated dose of 1 Gy.

### Biological processes regulated by miRNAs during IR treatment of chondrocytes differentiated from hiPSCs: genes involved in DDR are strongly controlled by the wide range of microRNAs

**Figure 7:**
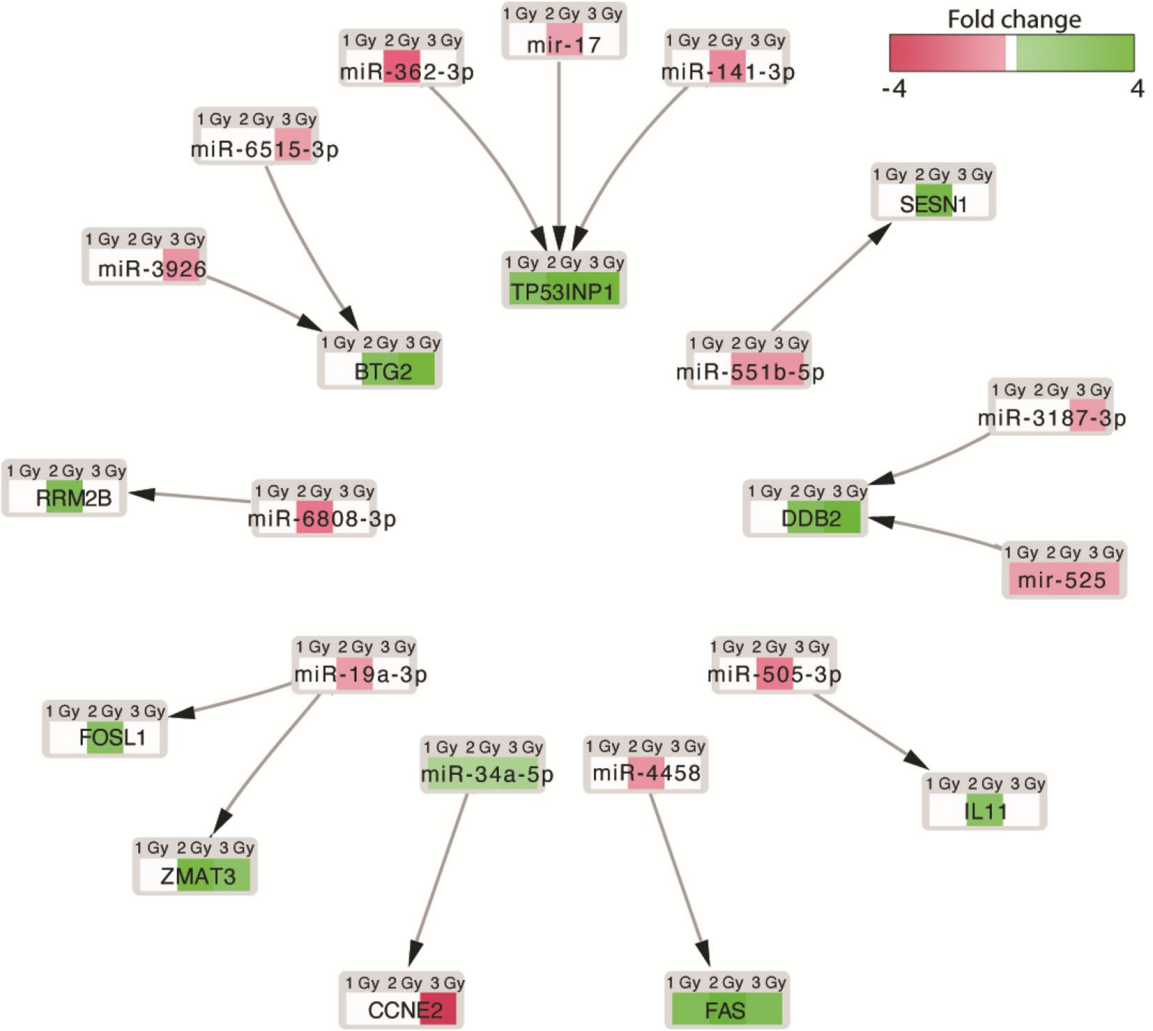
Interaction between IR treatment-related genes and their miRNAs (analyses based on microarray study). Validated target-miRNA pairs are negatively correlated.

We found that IR treatment-related genes were controlled by a range of different miRNAs, as follows: *CCNE2* (by hsa-miR-34a-5p); *ZMAT3* (hsa-miR-19a-3p); *FOSL1* (hsa-miR-19a-3p); *RRM2B* (hsa-miR-6808-3p); *BTG2* (hsa-miR-3926 and hsa-miR-6515-3p); *TP53INP1* (hsa-miR-362-3p, hsa-miR-17, and hsa-miR-141-3p); *SESN1* (hsa-miR-551b-5p); *DDB2* (hsa-miR-3187-3p and hsa-miR-525); *IL11* (hsa-miR-505-3p) and *FAS* (hsa-miR-4458) (Fig. 7). These genes are engaged in processes activated after IR (e.g., cell death: *ZMAT3*, *RRM2B*, *FAS*, *BTG2*, *TP53INP1*, *FOSL1*, *IL11*; DNA repair: *RRM2B*, *BTG2*, *DDB2*; signal transduction by p53 class mediator: *ZMAT3*, *RRM2B*, *BTG2*; and mitotic cell cycle: *CCNE2*).

The most upregulated and downregulated miRNAs (hsa-miR-19a-3p, hsa-miR-362-3p, and hsa-miR-34a-5p) were verified by RT-qPCR (Fig. 5).

### Analysis of the composition of the medium in chondrocyte-like cells differentiated from hiPSCs after IR treatment

One of the main aims of the present study was to assess the secretory activity of cells treated with IR and compare their activity level with non-irradiated cells. Thus, media from the cells were collected 24 h after IR. We found significant alterations in the following 38 pro- and anti-inflammatory cytokines: basic FGF; CTACK; Eotaxin; G-CSF; GM-CSF; GRO-alpha; HGF; IFN-alpha 2; IFN-gamma; IL-1 alpha; IL-1 beta; IL-2; IL-2R alpha; IL-4; IL-6; IL-7; IL-8; IL-9; IL-12; IL-13; IL-17; IP-10; LIF; MCP-1; MCP-3; M-CSF; MIG; MIP-1 alpha; MIP-1 beta; PDGF-BB; NGF-beta; RANTES; SCF; SCGF-beta; SDF-1 alpha; TNF-alpha; TNF-beta; and TRAIL) in the medium of hiPSC-derived cells after IR. By contrast, we did not observe any notable changes in the level of IL-3 and MIF. These results are shown in Table 1 as mean values (pg/ml of secreted cytokine) with standard deviation. The mean values ranged from 0.223 pg/ml (IL-3 in controls) to 3448.34 pg/ml (SCGF-beta in controls). In the graphs (Supplementary Fig. 3) we highlight the most statistically significant relationships between particular variants (controls vs. 1 Gy; controls vs. 2 Gy; controls vs. 3 Gy; 1 Gy vs. 2 Gy; 1 vs. 3 Gy and 2 Gy vs. 3 Gy). The statistical significance for each comparison is shown in Supplementary Table 1. Interestingly, in most cytokines, there was a notable decrease in secretion (e.g., G-CSF, GRO-alpha, HGF, IP-10, LIF, SCGF-beta, TNF-alpha) after 2 Gy irradiation. The cytokine secretion levels after 1 and 3 Gy were, to a large extent, more similar to that of non-irradiated cells than the 2 Gy dose (conventional therapy). These findings demonstrate that the specific cytokine secretion profile depends on the applied dose (and treatment protocol).

**Table 1:** A total of 38 pro- and anti-inflammatory cytokine secretion to the medium collected from hiPSC-derived cells (basic FGF, CTACK, Eotaxin, G-CSF, GM-CSF, GRO-alpha, HGF, IFN-alpha 2, IFN-gamma, IL-1 alpha, IL-1 beta, IL-2, IL-2R alpha, IL-4, IL-6, IL-7, IL-8, IL-9, IL-12, IL-13, IL-17, IP-10, LIF, MCP-1, MCP-3, M-CSF, MIG, MIP-1 alpha, MIP-1 beta, PDGF-BB, NGF-beta, RANTES, SCF, SCGF-beta, SDF-1 alpha, TNF-alpha, TNF-beta, TRAIL) significantly changed after IR.

Cytokine	Control		1 Gy		2 Gy		2 Gy	
	Mean (pg/ml)	SD	Mean (pg/ml)	SD	Mean (pg/ml)	SD	Mean (pg/ml)	SD
Basic FGF	19.648	5.273	21.490	2.179	18.333	3.029	24.775	1.437
CTACK	7.778	2.472	9.360	0.749	5.768	1.893	11.438	1.107
Eotaxin	0.973	0.256	1.180	0.270	0.340	0.307	1.448	0.249
G-CSF	314.197	28.588	254.550	48.708	201.782	10.046	359.657	27.892
GM-CSF	1.363	0.491	1.560	0.195	0.885	0.371	1.967	0.128
GRO-alpha	123.168	25.357	117.490	14.914	76.688	10.367	150.840	4.592
HGF	2312.825	177.547	1960.230	213.868	1207.147	411.302	2677.245	398.319
IFN-alpha 2	6.705	1.626	6.975	0.594	4.520	1.230	8.743	0.925
IFN-gamma	32.530	8.820	27.623	3.593	19.493	5.463	39.433	4.167
IL-1 alpha	6.745	1.183	11.013	2.636	10.698	11.200	13.440	0.815
IL-1 beta	1.598	0.472	1.817	0.382	1.020	0.414	2.110	0.250
IL-2	2.890	1.268	4.230	1.106	3.250	1.909	5.128	0.561
IL-2R alpha	6.285	2.236	7.258	0.954	4.650	1.802	9.245	0.972
IL-3	0.223	0.050	0.290	0.082	0.293	0.112	0.283	0.059
IL-4	7.515	2.248	7.300	0.254	5.090	0.393	9.755	0.168
IL-6	103.108	41.508	93.668	14.068	64.303	19.406	132.747	12.367
IL-7	3.800	1.462	7.825	1.694	9.005	0.926	7.338	0.879
IL-8	677.113	181.044	510.815	78.278	327.653	32.520	797.178	35.676
IL-9	9.873	1.523	12.588	1.227	5.738	2.262	15.790	0.703
IL-12	85.910	29.446	76.348	12.313	50.360	15.115	107.678	15.479
IL-13	0.643	0.299	1.008	0.106	1.075	0.0778	1.085	0.142
IL-17	4.275	1.160	5.533	0.587	7.310	3.199	6.065	0.367
IP-10	348.387	13.590	177.243	33.968	66.600	21.789	215.602	16.262
LIF	55.928	11.575	49.465	1.518	30.693	7.808	68.800	2.384
MCP-1	1734.480	637.348	1682.113	57.251	1072.748	331.551	1937.448	63.911
MCP-3	648.340	245.533	519.238	81.092	322.723	45.366	779.390	12.026
M-CSF	4.775	1.241	4.648	0.487	2.465	0.719	6.058	0.468
MIF	1268.858	146.577	1446.020	70.476	1201.818	297.113	1461.543	70.532
MIG	8.475	3.497	19.145	2.941	11.178	5.478	21.098	2.099
MIP-1 alpha	0.970	0.319	1.213	0.281	0.640	0.186	1.075	0,190
MIP-1 beta	10.583	5.196	19.758	10.370	6.823	1.852	11.310	4.546
NGF beta	17.983	5.930	21.703	2.334	12.658	1.154	29.325	0.924
PDGF-BB	22.680	7.070	24.740	3.225	14.783	3.941	31.728	4.881
RANTES	8.701	2.456	9.592	0.877	6.813	1.403	10.581	0.325
SCF	39.285	14.448	36.425	3.867	25.477	2.609	52.445	3.648
SCGF-beta	3448.343	153.597	2550.860	638.244	1258.005	382.718	3233.932	359.261
SDF-1 alpha	31.233	5.311	36.858	6.172	24.190	11.063	45.797	7.140
TNF-alpha	83.825	24.237	96.705	1.534	56.830	2.235	107.438	6.910
TNF-beta	9.330	2.040	9.302	2.051	4.048	0.972	11.572	0.577
TRAIL	462.770	174.123	440.743	81.543	249.300	29.693	641.723	21.068

The results are presented as mean values (pg/ml of secreted cytokine) with standard deviations.

## Discussion

Findings from many recent studies suggest that hiPSCs can differentiate into various types of functional cells such as cardiomyocytes and osteocytes^7^. However, transplantation of these cells into humans presents a major risk, as the data indicate that a small number of hiPSCs remain undifferentiated at transplantation and can spontaneously transform into rapidly proliferating tumors^14^.

Our group previously applied molecular methods to analyze DDR mechanisms in chondrocyte-like cells after IR^10^. In that study, we assessed, *inter alia,* the kinetics of DNA DSB formation, activation of DNA repair mechanisms, cell cycle changes, and apoptosis. Our findings showed that although hiPSC-derived chondrocyte cells readily formed DSBs and accumulated in the G2 phase, these cells were also characterized by highly efficient DNA repair mechanisms. Moreover, those cells appeared to be more likely to undergo senescence rather than apoptosis after IR exposure. However, in that study, we did not use conventional radiotherapy regimens/dose for IR^10^. Here, we also analyzed some important markers like proliferation activity (Supplementary Fig. 5), formation of DSBs (Supplementary Fig. 6) and necrosis level (Supplementary Fig. 7) in irradiated cells. Moreover, in the present study, we used a specially modified anthropomorphic phantom to ensure similar conditions to those present in the patient's larynx during treatment. This is an important advantage of the present study.

In this study, we focused on the high-throughput analysis of the response of chondrocyte-like cells differentiated from hiPSCs (Fig. 1 and Supplementary Fig. 4) to IR treatment based on hypofractionated and hyperfractionated protocols (Fig. 2). Our findings suggest that those cells are characterized by specific DDR mechanisms. Despite the immense promise of hiPSCs, data on the response of those cells to IR are limited, which is why we decided to investigate the gene and microRNA expression profile in hiPSC-derived chondrocytes after IR (Supplementary Fig. 1 and Supplementary Fig. 2). As noted above, since the gene expression profile of irradiated cells after 1 h did not reveal any significant changes (Supplementary Fig. 1), we only used the material collected 24 h after irradiation for all further analyses.

Below we discuss our findings in the context of the limited published data on the response of iPSC-derived cells and chondrocytes to IR.

Becker et al.^15^ irradiated hiPSC-derived cardiomyocytes with 5 Gy of x-ray irradiation and then performed deep-sequencing of RNA to investigate gene expression changes in these cells. Those authors identified genes implicated in cardiac calcium homeostasis (*PDE3B*), oxidative stress response (*FDXR* and *SPATA18*), and the etiology of cardiomyopathy (*SGCD*, *BBC3* and *GDF15*). The large fraction of downregulated genes was dominated by genes involved in cell cycle processes^15^. In our study, we also observed decreased expression of genes engaged in certain cell cycle processes in irradiated cells, including "cell cycle", "mitotic cell cycle process", "cell division" (Figs. 3, 4). Furthermore, we have demonstrated the close relationship between the most upregulated and downregulated genes (e.g., *TP53INP1*, *DDB2*, *ZMAT3, SESN1*) and the microRNAs that control their expression, such as hsa-miR-17, hsa-miR-5252, hsa-miR-19a-3p, and hsa-miR-551b-5p (Figs. 5, 7).

Miyake et al.^16^ successfully derived keratinocytes from iPSCs and characterized the differentiated states and DDR of these cells. The derived keratinocytes showed progenitor-like characteristics (e.g., high expression of integrin a6/CD71 and resistance to IR) as a result of DDR. After IR exposure, g-H2AX foci decreased more slowly in passages 2 and 3 keratinocytes compared to passage 1 keratinocytes, which suggests that matured cells attenuate DNA repair activity^17^.

Hong et al. showed that low-dose γ-radiation (LDR)^19^ (0.5–1 cGy) suppresses IL‐1β‐induced dedifferentiation and inflammation of chondrocytes without any noticeable side effects. In that study, LDR attenuated signaling pathways both upstream and downstream of catenin, blocked dedifferentiation process throughout preventing IL‐1β‐induced Sox‐9 suppression, and inhibited inflammation by counteracting IL‐1β‐induced activation of NF‐κB. These anti‐disease effects establish LDR as a potentially valuable therapeutic tool for patients with cytokine‐mediated cartilage disorders^19,20^.

Lepleux et al.^18^ found that the radiation-induced bystander response can be transferred from chondrosarcoma cells treated with IR to non-irradiated chondrocyte cells. A great impact on chondrocyte survival was observed at low doses. Besides, this effect was more notable after low LET (X-rays) in comparison with high LET irradiation (C-ions). Bystander factors secreted in the conditioned medium contributed to the reduced proliferation and elevated DNA damage level at low doses (X-rays and C-ions). Bystander biological activity was absent after dilution and heat treatment of the conditioned medium, leading the authors to suggest that factors such as TNF-α and IL-6 may have contributed to this effect^18^.

The findings of the present study, based on TCGA data, indicate that the downregulated expression of genes engaged in the cell cycle and cell division in hiPSC-derived chondrocytes differed from the gene expression profile of tumor and normal tissue samples from laryngeal tissues, in which those genes were strongly upregulated. Similarly, the differentiated cells presented a dose-dependent upregulation of some genes involved in DDR compared to laryngeal tumor cells (Fig. 6). This negative correlation suggests that DDR mechanisms in hiPSC-derived chondrocytes may be more similar to differentiated mature cells than to potentially tumorigenic hiPSCs or partially differentiated cells. We assume that irradiation of these cells (hiPSC-derived chondrocytes) is unlikely to induce malignant transformation; consequently, it should be safe to use such cells. However, more research is needed to elucidate this poorly understood area.

In general, inflammatory cytokines, GFs, and proteases can affect cancer cell invasion, the bystander effect, and radiation-derived tissue complications including fibrosis and genomic instability. As a result, these factors can greatly impact intrinsic cellular radiosensitivity^21^. IR can provoke a multi-layered signaling response by activating many pro-survival pathways accompanied by transient activation of key transcription factors such as Nuclear Factor kappa B (NF-ĸB) as well as signal transducers and activators of transcription members (STATs)^22^. NF-ĸB plays a crucial role in immune and inflammatory responses due to its ability to regulate the expression of proinflammatory cytokines and chemokines such as tumor necrosis factor-alpha (TNF-α), interleukin-1 (IL-1), IL-2, IL-6, and monocyte chemoattractant protein-1 for inflammatory cells (MCP-1)^23^.

There is evidence^24,25^ that exposure of cells and tissues to IR stimulates the expression of many different cytokines and GFs, including the following: TNF-α, interleukin-1 alpha (IL-1α), interleukin-1 beta (IL-1β), IL-6, type I interferon (type I IFN), granulocyte–macrophage colony-stimulating factor (GM-CSF), IL-4, IL-5, IL-10, IL-12, IL-18, and TGF-β. The inflammatory reaction induced by IR is mediated by inflammation-related cytokine genes such as TNF-a, IL-1, IL-6, IL-8, interferon-gamma (IFN-γ), granulocyte–macrophage colony-stimulating factor (G-CSF), vascular endothelial growth factor (VEGF), and epidermal growth factor receptor. Additionally, downregulation of the existing inflammatory response depends largely on the half-life of the proinflammatory cytokines and the production of the antiinflammatory cytokines, such as IL-4, IL-10, IL-13, and TGF-β^24,25^. Similarly, Schaue et al.^26^ found that in vitro and in vivo exposure of cells and tissues to IR treatment induces expression of many cytokines and GFs, including the following: TNF-a, IL-1aa, IL-1b, type I IFN, GM-CSF, IL-4, IL-5, IL-6, IL-10, IL-12, IL-18, VEGF, bFGF, and TGF-βa. Many of these appear as immediate early genes and thus qualify as “radiation-inducible”. In tissues, radiation damage ultimately manifests as fibrosis. Macrophages accumulate in the damaged tissue and subsequently switch from being proinflammatory to producing fibrosis-promoting cytokines (PDGF and TGF- β)^26^. The radioprotective effect of IL-12 in hematopoietic tissue depends on its interaction with IL-1 and SCF. Whereas, the sensitizing effect on gastrointestinal tissue is mediated by IFN-y and TNF^27^.

Schrӧder et al.^28^ found that IR causes nonlinear dose-dependent effects on the secretion of proinflammatory cytokines. Among the 23 inflammatory markers assessed in that study, four of them: KC, MCP-1, RANTES, and G-CSF were found to be significantly altered. The findings of that study indicate that LDR has an immune modifying capacity on the response of endothelial cells, both in a conventional 2D culture system and an ECM-based 3D model^28^.

In a mouse model, Shan et al.^29^ described the effect of various doses (range, 0.05–6 Gy) of whole-body irradiation (WBI) on peritoneal macrophages. Those authors found a marked stimulation of IL-12 and IL-18 secretion correlated with the nuclear translocation of NF-ĸB. Moreover, upregulated expression of cytoplasmic MyD88 and the surface molecules CD14 and TLR4-MD2 was also proved. The authors also demonstrated stimulation of IL-12 and IL-18 secretion by mouse peritoneal macrophages after WBI. They explored the possible mechanisms and implications of these findings in cancer radiotherapy. Both low (0.075 Gy) and high (2 Gy) dose IR led to sustained stimulation of IL-12 and IL-18 secretion by mouse macrophages. This finding was paralleled by activation of NF-ĸB as well as upregulated expression of CD14 and TLR4–MD2 on the macrophage surface and MyD88 in the cytoplasm^29^.

Kiang et al., based on cytokine profiling of blood from B6D2F1 female mice irradiated at low dose rates, selected G-CSF and IL-18 for the extended experiment involving higher dose rates and large dose range. Those authors found that radiation dose rates do not influence the cytokine response to IR. Moreover, G-CSF responded to all of the tested radiation dose rates, which ranged from 0.04 Gy/min to 1.94 Gy/min. The increases were biphasic, occurring on day 2. In contrast to G-CSF, circulating IL-18 increased later and in a persisted manner^30^.

Our findings also show that cytokines play a key role in the DDR of irradiated cells. We found 38 cytokines that differed significantly after IR (Table 1 and Supplementary Fig. 3). We observed altered secretion of numerous relevant cytokines, including IL-12, IL-6, IL-8, HGF, MCP-3, TNF-beta, TRAILGRO-alpha, and IFN-gamma. Interestingly, after irradiating cells with 2 Gy of IR, the secretion level of most cytokines decreased, which may partly explain why 2 Gy is commonly used in conventional radiotherapy: it provides an effective, therapeutic fractional dose but does not induce an excessive immune response.

## Conclusions

In the present study, we have demonstrated, in a purpose-designed phantom, the broad context of activated DDR mechanisms of irradiated hiPSC-derived chondrocytes. The activation of crucial signaling pathways following IR was notable, with both upregulation and downregulation of a wide range of genes involved in DDR. Our analysis revealed that expression of those genes is regulated by specific microRNAs. Based on proteomics, we identified the profile of 38 cytokines secreted into the medium after IR. We believe the findings reported here help explain how stem cell-derived cells respond to irradiation, findings that may have clinically significant implications. Our results show that changes in both the mRNA and microRNA expression profiles become increasingly evident as the fractional radiation dose increases (hyperfractionation < conventional therapy < hypofractionation). However, a fractional dose of 2 Gy—the conventional radiotherapy dose—does not cause excessive activation of immune response components. Finally, based on TCGA data, it appears that irradiation of chondrocyte-like cells is unlikely to lead to malignant transformation, which suggests that they can be safely used for transplantation purposes.

## Materials and methods

### Cell culture

The commercially available hiPSC cell line ND41658*H (Coriell Cell Repository, NY, USA) was cultured as described elsewhere^31^.

HiPSCs were used to form EBs. After 7 days, the EBs were differentiated into a chondrogenic lineage in a defined medium supplemented with transforming growth factor-beta (TGF-β) (10 ng/ml) according to a previously established protocol^32^ (Fig. 1).

### Irradiation and dosimetry

To irradiate the chondrocyte-like cells, the specially modified and redesigned Alderson Radiation Therapy phantom (ART) was used to obtain conditions similar to those present in the larynx of a patient with laryngeal cancer. It is crucial to establish proper attenuation of radiation (total mass attenuation coefficients similar to those present in the patient’s tissue, adequate energy spectrum, absorbed and scatter radiation to the cell layer). The ART phantom was immobilized with the QFixEncompassTM system (Avondale, PA, USA) to ensure positioning reproducibility during preparation and irradiation procedure. Tomographic scans of the phantom were performed using Siemens SOMATOM Definition AS (120 kV X-Ray Tube Voltage, 1 mm slice thickness, head, and neck protocol).

An Eppendorf tube with 1 × 10^6^ chondrocyte-like cells was used to irradiate the cells. The quasi-gross tumor volume (GTV) was contoured. Geometrical margins of 1 cm around the GTV were applied to create the quasi-clinical target volume (CTV). Following typical procedures for H&N cancer, a 0.5 cm margin around the CTV was added to ensure that the quasi-PTV received the prescribed doses. In the phantom, we also outlined quasi-OARs representing the spinal cord, parotid glands, and mandible. Treatment plans were prepared using the volumetric modulated arc therapy (VMAT) technique with two co-planar arcs (Fig. 2). The goal of the plans was to cover at least 95% of the PTV with the planned prescription dose (1, 2, or 3 Gy). Optimization and calculations were done on the Eclipse Planning System, v. 15.6 (Varian Medical Systems, USA) using the anisotropic analytical algorithm (AAA).

Irradiation was performed with a 6 MV photon beam produced by the Unique linac (Varian Medical Systems, USA) with a 120 leaf multileaf collimator and a maximal nominal dose rate of 600 MU/min. Phantom positioning was done using the X-ray 6D IGRT (orthogonal kV-kV) imaging system with the BrainLabExacTrac (BrainLab AG, Feldkirchen, Germany). Before irradiation, the electronic portal imaging device (EPID) system was used to perform dosimetric pre-verification. For the gamma evaluation criteria 3%/3 mm, the following results were obtained for the plans (%): 1 Gy: 98.3, 98.2; 2 Gy: 99.3, 98.8; and 3 Gy:, 96.4, 98.1 (Fig. 2).

### Immunofluorescence analysis and alcian blue staining

HiPSC-derived chondrocytes were stained with the use of following antibodies: anti-type II collagen, aggrecan and anti-cartilage oligomerix matrix protein (COMP) according to a previously established protocol^32^.

Alcian blue staining was performed as previously described^33^ to assess the deposition of proteoglycans in the differentiated cells.

### Microarray study

Total RNA and miRNA-enriched fraction from hiPSCs after chondrogenic differentiation was purified with the miRNeasy Kit and RNeasy MinElute according to the manufacturer’s instructions (217004, Qiagen, Hilden, Germany). The microarray study was conducted according to procedures described elsewhere^34–36^. The obtained CEL files were further analyzed using the R statistical language and Bioconductor package, including the relevant Bioconductor libraries. For the normalization, background correction, and calculation of the expression values of the examined genes or microRNAs, the Robust Multiarray Average (RMA) normalization algorithm implemented in the “Affy” library was applied. A complete gene/microRNA data table involving normalized gene/microRNA expression values, gene/microRNA symbols, gene/microRNA names and Entrez IDs was generated on the basis of assigned biological annotations taken from the “pd.hugene.2.1.st” and “pd.mirna3.1” libraries. For the expression and statistical assessment, the linear models for microarray data included in the “limma” library was applied. The established cut-off criteria were based on both differences in expression fold change (FC) greater than abs. 1.5 and an adjusted p value of ≤ 0.05. Genes/microRNAs fulfilling the aforementioned selection criteria were considered to be significantly different and therefore were subjected to the further analyses.

### Real-time quantitative polymerase chain reaction (RT-qPCR) evaluation

Real Time-PCR reactions were performed using the PrimePCR™ Custom Plates (Bio-Rad, CA, USA) and the specific synthesized primers for each gene: *CCNE2*, *DLGAP5*, *KIF20B*, *FAS*, *TP53INP1*, *BTG2*, *DDB2*, *ZMAT3* and *CENPE* cDNA samples were analyzed for genes of interest and the reference gene beta-2-microglobulin (B2M). The expression level for each target gene was calculated as − 2ΔΔC_t_. The reaction was performed in triplicate for the gene of interest.

### Gene expression profile of miRNAs and miRNA-Target gene prediction

Profiling of miRNA expression was conducted using Affymetrix platform-based microarrays with GeneChip™ miRNA 4.1 Array Strip (Thermo Fisher Scientific, Waltham, MA, USA), as previously described^37^. To identify potential target genes for differentially expressed miRNA, we took advantage of a SpidermiR package. Differentially expressed miRNAs were used as a query for searching target genes in the following databases: For predicted targets—DIANA, Miranda, PicTar, TargetScan, and for experimentally validated targets—miRTar, miRWalk.

All raw data files and a technical description were deposited in the gene expression omnibus (GEO) repository at the National Center for Biotechnology Information (http://www.ncbi.nlm.nih.gov/geo/) under the GEO accession number: GSE158073.

### Validation of miRNA expression

To validate differentially expressed microRNAs (hsa-miR-19a-3p, hsa-miR-362-3p, hsa-miR-34a-5p) selected from microarray studies, isolated microRNA from irradiated hiPSC-derived chondrogenic progenitors were reverse transcribed with TaqMan™ Advanced miRNA cDNA Synthesis Kit (ThermoFisher Scientific, MA, USA). Quantitative real-time PCR analysis was performed with TaqMan™ Fast Advanced Master Mix (ThermoFisher Scientific, MA, USA) and TaqMan™ Advanced miRNA Assay, specific to the selected miRNA. The hsa-miR-361-5p microRNA (ThermoFisher Scientific, MA, USA) served as an endogenous internal control and was used to normalize RT-qPCR results.

### Analysis of medium composition

The composition of the medium was analyzed on the Bio-Plex 200 system (BioRad Laboratories, Hercules, CA, USA). We used the Bio-Plex Pro Human Cytokine 48-plex to analyze cytokines, chemokines, and growth factors (GF) potentially involved in the inflammatory process and in the initiation and progression of cancer. The concentrations (pg/ml) of these molecules were compared to the standard curve of serial dilutions of standard molecules. The analysis was performed according to the manufacturer’s instructions. Each sample was analyzed in duplicate.

### RTCA cell proliferation assay

To verify the effect of IR on the proliferation rate of chondrocyte-like cells differentiated from hiPSCs, we applied an electrical impedance-based cell proliferation assay, named Real-Time Cell Analyser (RTCA, Roche Applied Science, GmbH, Penzberg, Germany)^38^. The RTCA system detects fluctuations in electrical impedance on the integrated sensory electrodes located at the bottom of the chamber's 16-hole slide plates (E-Plate 16), which are covered by dividing cells. Electrical impedance is measured at 15-min intervals throughout the cultivation period. The main RTCA readout is the “Cell Index”—a measurable parameter corresponding to the relative change in electrical impedance depending on the rate of proliferation or apoptosis of the cultivated cells. Each experimental group was seeded in the eight E-plate wells to a final volume of 200 μl per well. The cell index was normalized (normalized cell index) at time of examined substances administration using software from the same manufacturer (RTCA Software, v. 1.2, November 2009)^38^. Irradiated cells were cultivated per approximately 48 h. Each experiment was repeated ≥ three times. We decided to present results involving proliferation completed in 39 h because after this time, the control (0 Gy) reached a plateu.

### Flow cytometry analysis

24 h after irradiation, cells were stained for γH2AX with the Alexa Fluor 647 Mouse Anti-H2AX (pS139) (560,447, BD Biosciences, NJ, USA) and for PerCP-Cy^TM^5.5 Mouse Anti-BrdU antibodies (51–9,007,682, BD Biosciences, NJ, USA). All procedures were carried out according to the manufacturer’s instructions. The procedure of necrosis measurement involved the incubation of cells with propidium iodide at 4 °C for 30 min. Cells were resuspended in 1 ml staining buffer and analyzed with a flow cytometer (CytoFlex, Beckman Coulter, CA, USA). Fluorescence intensity in arbitrary units was plotted in histograms and density plots. Data were analyzed using FlowJo software (FlowJo v10; LLC, Ashland, OR, USA). In the case of necrosis analysis, as an additional experiment we investigated the response of chondrocyte-like cells obtained via another chondrogenic protocol to IR. Those cells were obtained via differentiation in vitro in monolayer culture (Directed)^32^ in contrast to cells generated from differentiation via EBs in the presence of TGF-β3 (TGF-β3).

### Statistical analysis

Except for microarray analysis, all the other data were statistically analyzed using the Graphpad software (version 5.0; GraphPad Software, Inc., La Jolla, CA, USA). All experiments were performed at least 3 times. Results are presented as means ± standard deviation Differences between two groups and differences between multiple groups were identified by Student’s t-test and analysis of variance (ANOVA), respectively. *p* < 0.05 was considered statistically significant. The present study was supported by the National Science Centre (Grant No. 2016/23/N/NZ7/01,892) (recipient: ES) and Greater Poland Cancer Centre (Grant No. 7/02/2020/PRB/WCO/001) (recipient: ES).

## Supplementary Information

Supplementary Information 1.
