# Supplementary material for: Ionizing radiation exposure of stem cell-derived chondrocytes affects their gene and microRNA expression profiles and cytokine production
Source: Sci Rep. 2021 Apr 5;11:7481. doi: 10.1038/s41598-021-86230-1 (PMC8021574; doi:10.1038/s41598-021-86230-1)
Supplement: Supplementary file 1 — Supplementary Information 1. [file 41598_2021_86230_MOESM1_ESM.docx]

**Ionizing radiation exposure of stem cell-derived chondrocytes affects their gene and microRNA expression profiles and cytokine production**

Ewelina Stelcer^1,2,3*^, Katarzyna Kulcenty^2,1^, Marcin Rucinski^3^, Marta Kruszyna-Mochalska^1,4^, Agnieszka Skrobala^1,4^, Agnieszka Sobecka^2,5^, Karol Jopek^3^, Wiktoria Maria Suchorska^2,1^

^1^Department of Electroradiology, Poznan University of Medical Sciences, Garbary 15th, 61-866 Poznan, Poland

^2^Radiobiology Lab, Greater Poland Cancer Centre, Garbary 15th Street, 61-866 Poznan, Poland

^3^Department of Histology and Embryology, Poznan University of Medical Sciences, Swiecickiego 6 Street, 60-781 Poznan, Poland

^4^Department of Medical Physics, Greater Poland Cancer Centre, Garbary 15th, 61-866 Poznan, Poland

^5^Department of Head and Neck Surgery, Poznan University of Medical Sciences, Garbary 15th, 61-866 Poznan, Poland

*Corresponding author

[ewelina.stelcer@wco.pl](mailto:ewelina.stelcer@wco.pl)


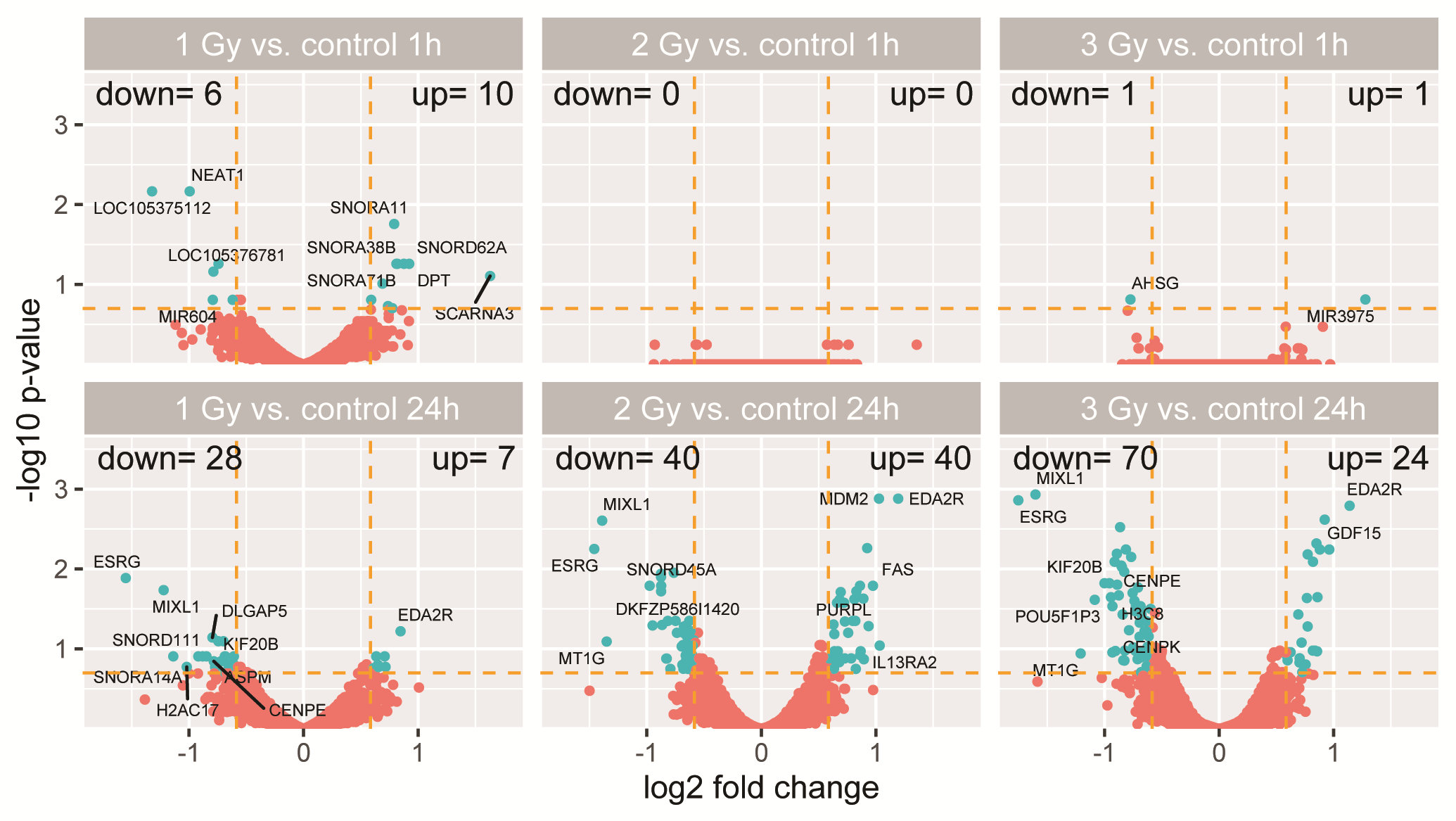


Supplementary Figure 1. Volcano plots displaying the total gene expression profile of the hiPSCs cells after IR compared to untreated controls. Each dot corresponds to the mean expression value of a specific gene. Dotted lines indicate cut-off values (1.5-fold change in expression and p<0.05 with 20% FDR correction). Dotted lines denote cut-off values. The red dots show the genes below the cut-off limit. The turquoise colour refers to genes that are either upregulated or downregulated. The ten genes with the greatest differences in fold change values are marked with the appropriate gene symbols.


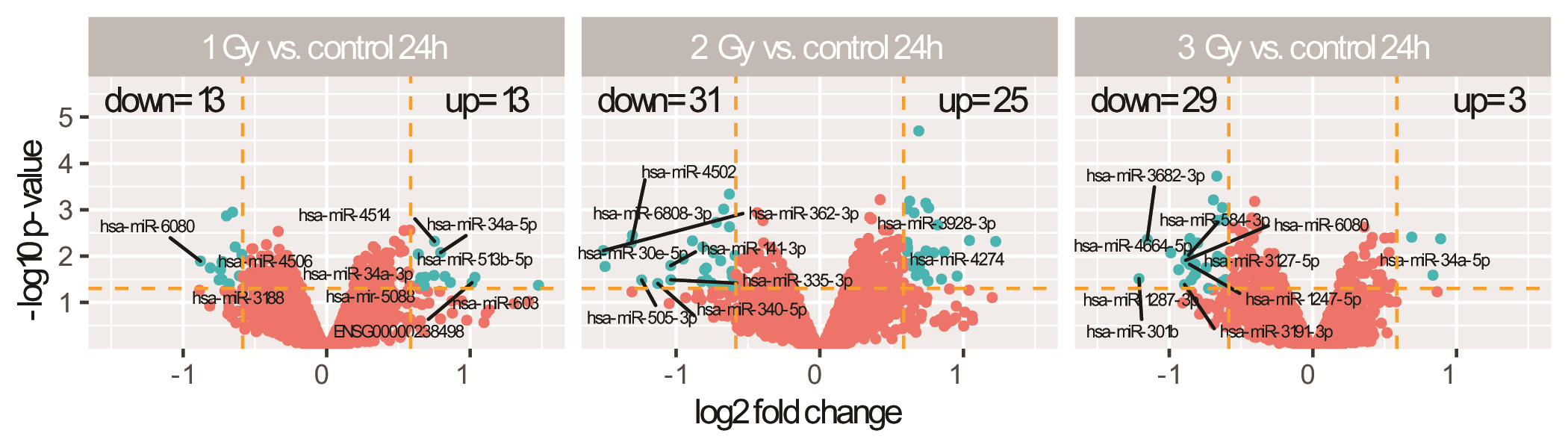


Supplementary Figure 2. Volcano plots of the total miRNA expression profile of the hiPSCs cells after IR compared to untreated controls. All markings are the same as in Supplementary Figure 1.


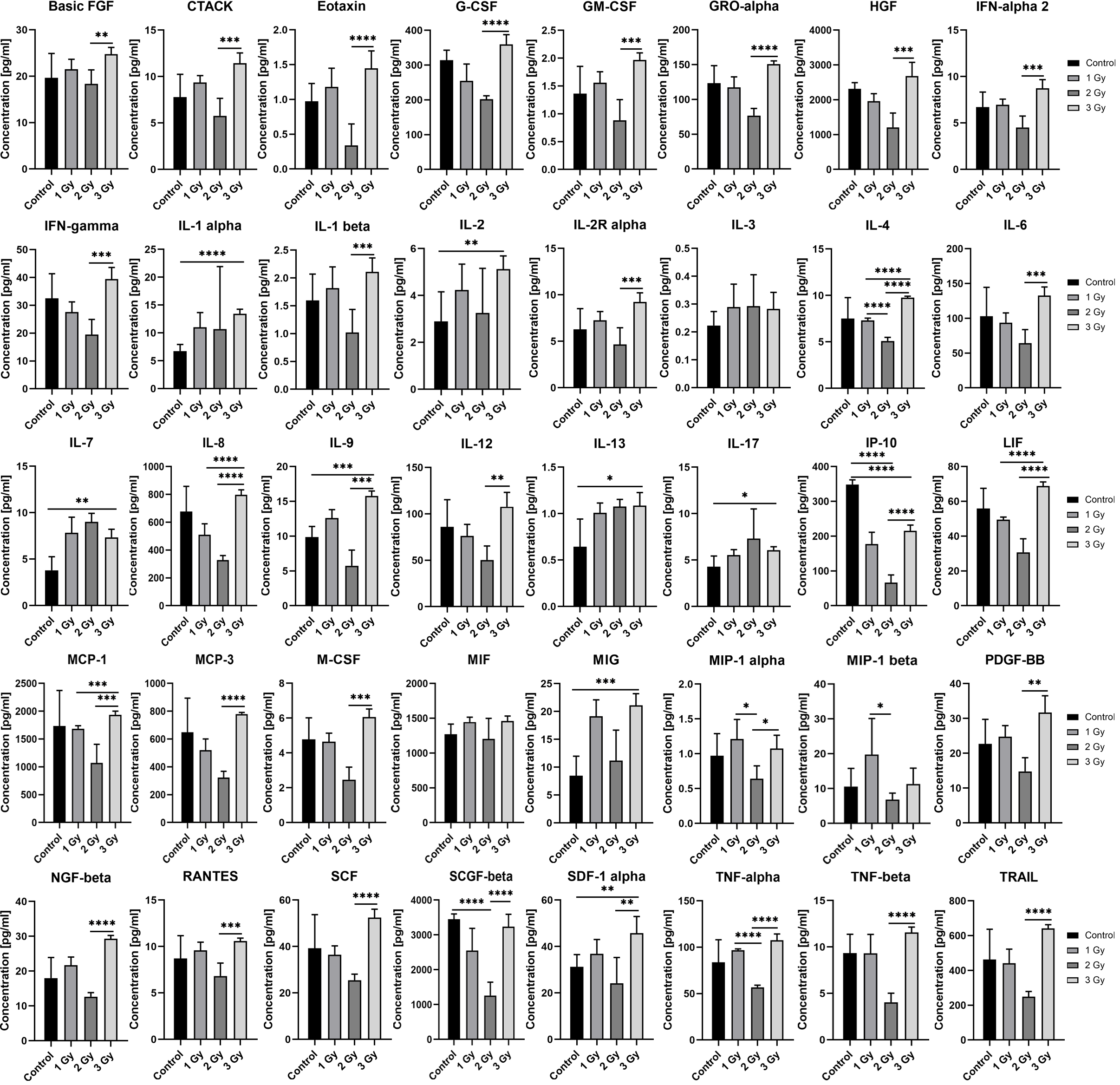


Supplementary Figure 3. Media from the cells were collected 24 h after IR treatment. This revealed 38 pro- and anti-inflammatory cytokines including the following: basic FGF, CTACK, Eotaxin, G-CSF, GM-CSF, GRO-alpha, HGF, IFN-alpha 2, IFN-gamma, IL-1 alpha, IL-1 beta, IL-2, IL-2R alpha, IL-4, IL-6, IL-7, IL-8, IL-9, IL-12, IL-13, IL-17, IP-10, LIF, MCP-1, MCP-3, M-CSF, MIG, MIP-1 alpha, MIP-1 beta, PDGF-BB, NGF-beta, RANTES, SCF, SCGF-beta, SDF-1 alpha, TNF-alpha, TNF-beta, and TRAIL. These were all significantly changed in the medium contained irradiated hiPSC-derived cells.


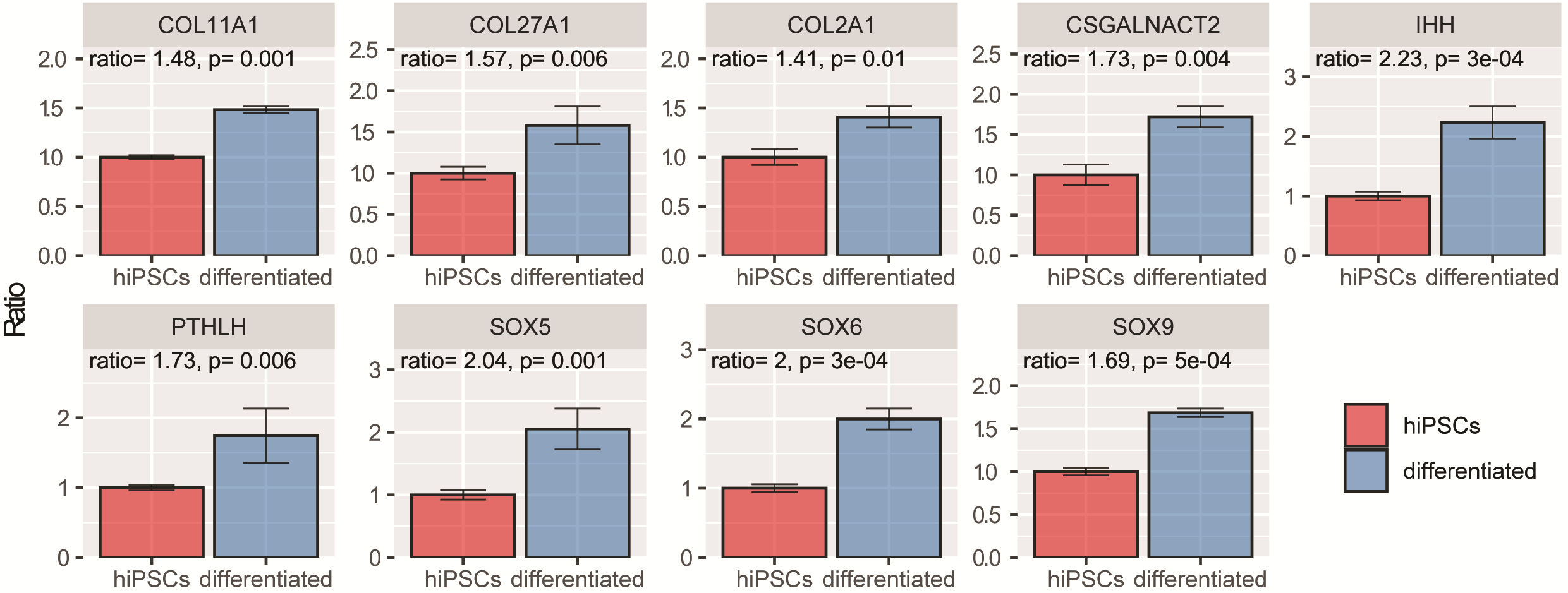


Supplementary Fig. 4. Based on microarray data, we compared transcriptome of hiPSCs before and after chondrogenic differentiation *in vitro*. We proved that hiPSC-derived chondrocytes reveal elevated level of expression of genes related to chondrogenesis: *COL11A1*, *COL27A1*, *COL2A1*, *CSGALNACT2*, *IHH*, *PTHLH*, *SOX5*, *SOX6*, and *SOX9*.


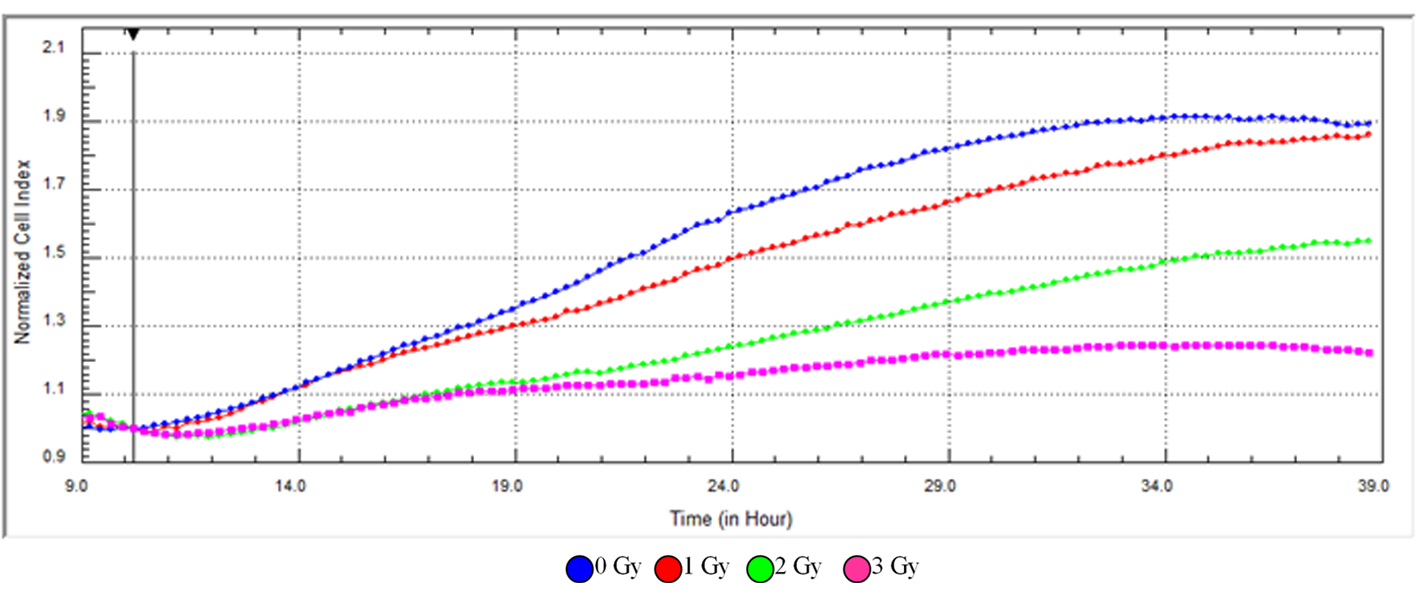


Supplementary Fig. 5. Effect of IR on the proliferation rate of irradiated chondrocyte-like cells using xCelligence Real-Time Cell Analyzer–RTCA. The RTCA chart presents mean normalized cell index. As the dose increases, the proliferative activity of irradiated cells decreases.


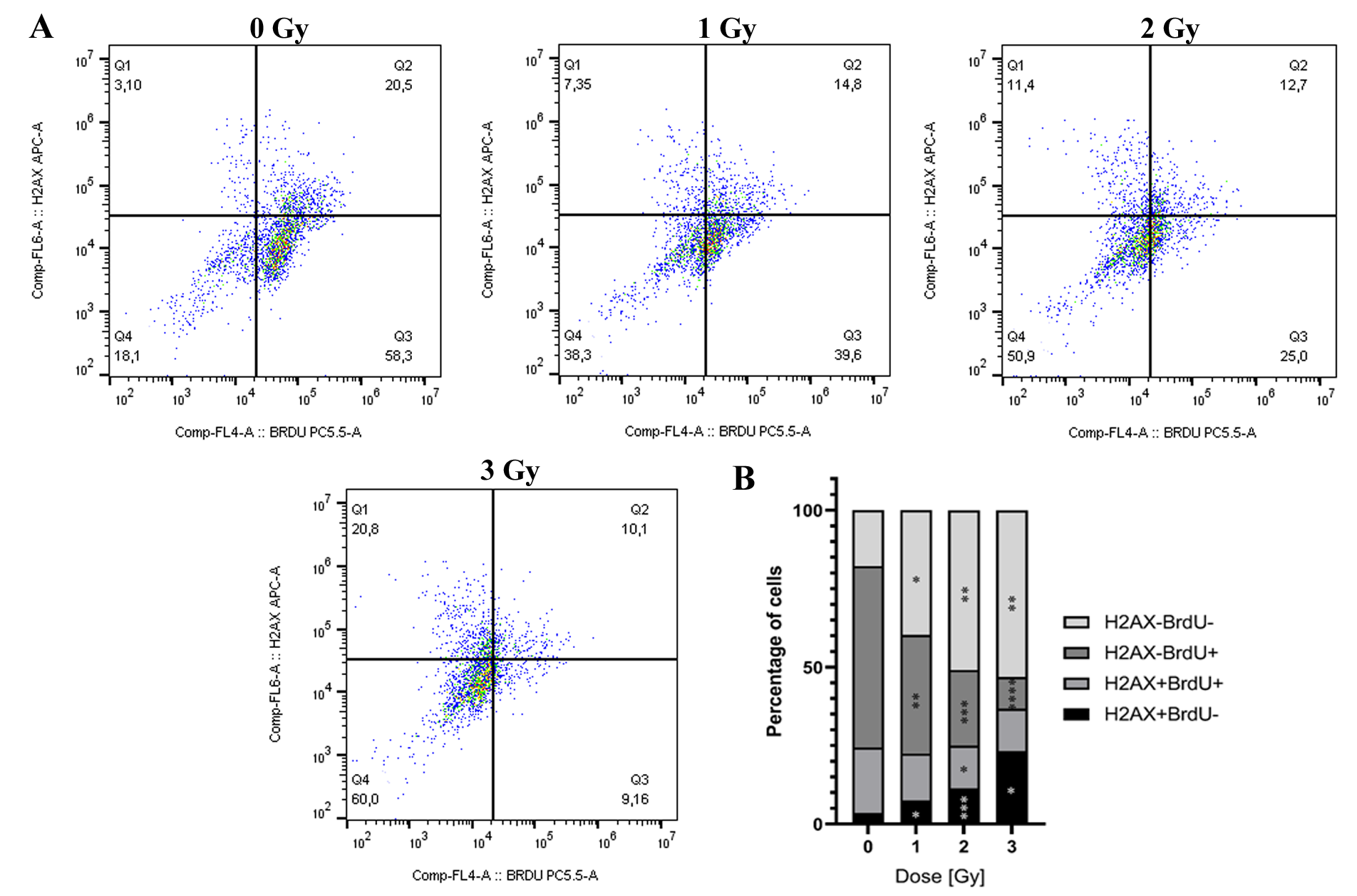


Supplementary Fig. 6. The analysis of phosphorylated H2AX and incorporated Bromodeoxyuridine (BrdU) revealed that IR causes increase in DSBs visualized as γH2AX and reduction in proliferation activity (BrdU) of examined cells (24h after IR treatment) (A). The most notable and statistically significant changes were observed in 3 Gy vs control (B).


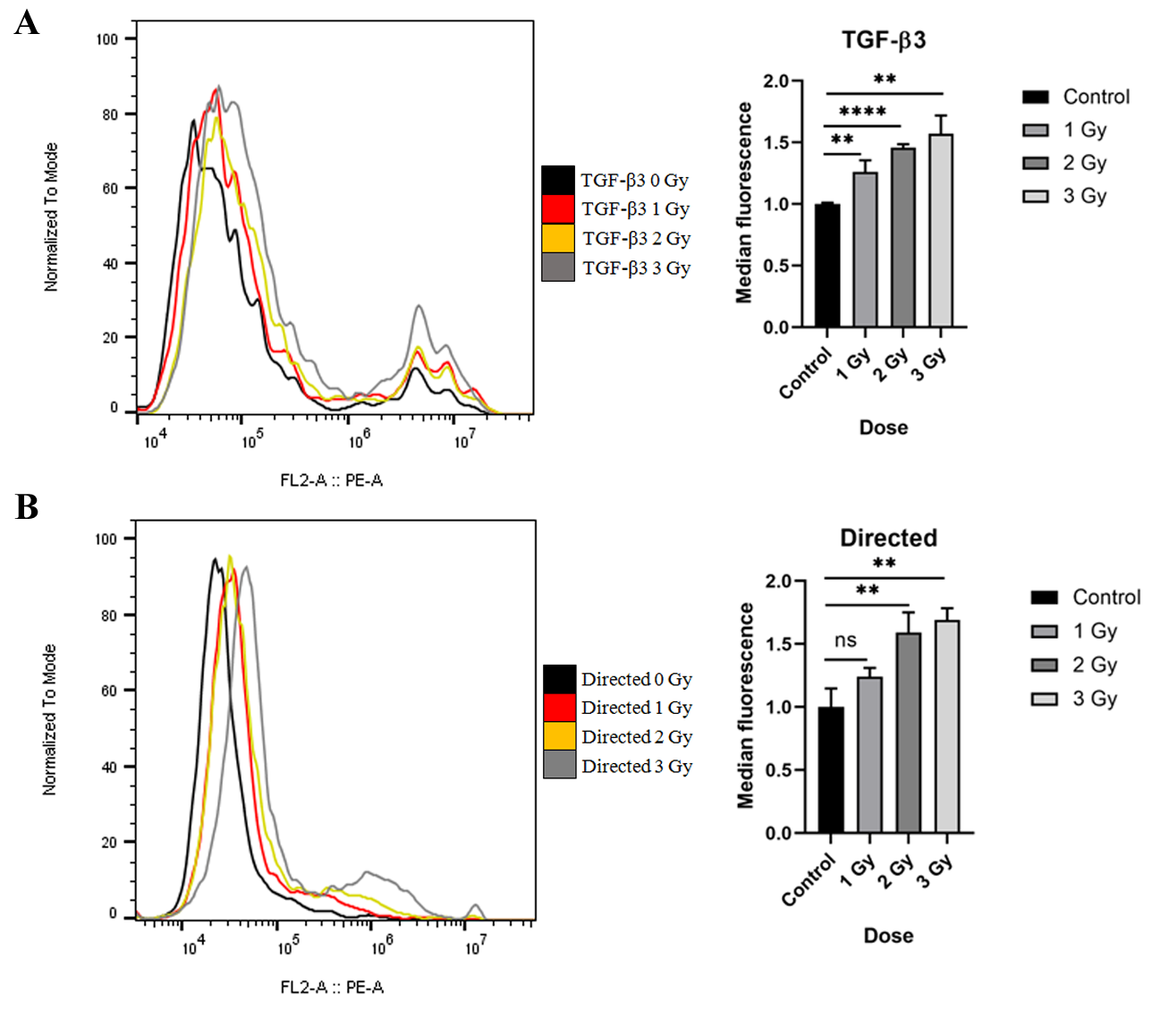


Supplementary Fig. 7. Both chondrocyte-like cells differentiation from hiPSCs: generated via EBs in the presence of TGF-β3 (A) and via monolayer 3-week culture (B) led to the increase of necrosis level after IR. The increase of the number of necrotic cells 24h after IR, was strictly correlated with the applied dose.

Supplementary Table 1. Overview of all statistically significant comparisons between particular variants (control vs 1 Gy; control vs 2 Gy; control vs 3 Gy; 1 Gy vs 2 Gy; 1 vs 3 Gy and 2 Gy vs 3 Gy) in terms of cytokine levels.

| G-CSF | |
| --- | --- |
| variant | Statistical significance |
| Control vs. 1 Gy | ns |
| Control vs. 2 Gy | *** |
| Control vs. 3 Gy | ns |
| 1 Gy vs. 2 Gy | * |
| 1 Gy vs. 3 Gy | ** |
| 2 Gy vs. 3 Gy | **** |

| GRO-alpha | |
| --- | --- |
| variant | Statistical significance |
| Control vs. 1 Gy | ns |
| Control vs. 2 Gy | * |
| Control vs. 3 Gy | * |
| 1 Gy vs. 2 Gy | ** |
| 1 Gy vs. 3 Gy | *** |
| 2 Gy vs. 3 Gy | **** |

| IP-10 | |
| --- | --- |
| variant | Statistical significance |
| Control vs. 1 Gy | *** |
| Control vs. 2 Gy | **** |
| Control vs. 3 Gy | **** |
| 1 Gy vs. 2 Gy | ** |
| 1 Gy vs. 3 Gy | * |
| 2 Gy vs. 3 Gy | **** |

| TNF-alpha | |
| --- | --- |
| variant | Statistical significance |
| Control vs. 1 Gy | ns |
| Control vs. 2 Gy | ns |
| Control vs. 3 Gy | * |
| 1 Gy vs. 2 Gy | **** |
| 1 Gy vs. 3 Gy | * |
| 2 Gy vs. 3 Gy | **** |

| LIF | |
| --- | --- |
| variant | Statistical significance |
| Control vs. 1 Gy | ns |
| Control vs. 2 Gy | * |
| Control vs. 3 Gy | * |
| 1 Gy vs. 2 Gy | ** |
| 1 Gy vs. 3 Gy | **** |
| 2 Gy vs. 3 Gy | **** |

| IL-8 | |
| --- | --- |
| variant | Statistical significance |
| Control vs. 1 Gy | ns |
| Control vs. 2 Gy | ** |
| Control vs. 3 Gy | ns |
| 1 Gy vs. 2 Gy | *** |
| 1 Gy vs. 3 Gy | **** |
| 2 Gy vs. 3 Gy | **** |

| IL-12 | |
| --- | --- |
| variant | Statistical significance |
| Control vs. 1 Gy | ns |
| Control vs. 2 Gy | ns |
| Control vs. 3 Gy | ns |
| 1 Gy vs. 2 Gy | * |
| 1 Gy vs. 3 Gy | * |
| 2 Gy vs. 3 Gy | ** |

| HGF | |
| --- | --- |
| variant | Statistical significance |
| Control vs. 1 Gy | ns |
| Control vs. 2 Gy | * |
| Control vs. 3 Gy | ns |
| 1 Gy vs. 2 Gy | * |
| 1 Gy vs. 3 Gy | * |
| 2 Gy vs. 3 Gy | *** |

| IFN-gamma | |
| --- | --- |
| variant | Statistical significance |
| Control vs. 1 Gy | ns |
| Control vs. 2 Gy | * |
| Control vs. 3 Gy | ns |
| 1 Gy vs. 2 Gy | * |
| 1 Gy vs. 3 Gy | ** |
| 2 Gy vs. 3 Gy | *** |

| IL-6 | |
| --- | --- |
| variant | Statistical significance |
| Control vs. 1 Gy | ns |
| Control vs. 2 Gy | ns |
| Control vs. 3 Gy | ns |
| 1 Gy vs. 2 Gy | * |
| 1 Gy vs. 3 Gy | ** |
| 2 Gy vs. 3 Gy | *** |

| MCP-1 | |
| --- | --- |
| variant | Statistical significance |
| Control vs. 1 Gy | ns |
| Control vs. 2 Gy | ns |
| Control vs. 3 Gy | ns |
| 1 Gy vs. 2 Gy | * |
| 1 Gy vs. 3 Gy | *** |
| 2 Gy vs. 3 Gy | *** |
| MCP-3 | |
| variant | Statistical significance |
| Control vs. 1 Gy | ns |
| Control vs. 2 Gy | * |
| Control vs. 3 Gy | ns |
| 1 Gy vs. 2 Gy | ** |
| 1 Gy vs. 3 Gy | *** |
| 2 Gy vs. 3 Gy | **** |

| MIF | |
| --- | --- |
| variant | Statistical significance |
| Control vs. 1 Gy | ns |
| Control vs. 2 Gy | ns |
| Control vs. 3 Gy | ns |
| 1 Gy vs. 2 Gy | ns |
| 1 Gy vs. 3 Gy | ns |
| 2 Gy vs. 3 Gy | ns |

| TRIAL | |
| --- | --- |
| variant | Statistical significance |
| Control vs. 1 Gy | ns |
| Control vs. 2 Gy | ns |
| Control vs. 3 Gy | ns |
| 1 Gy vs. 2 Gy | ** |
| 1 Gy vs. 3 Gy | ** |
| 2 Gy vs. 3 Gy | **** |

| SCGF-beta | |
| --- | --- |
| variant | Statistical significance |
| Control vs. 1 Gy | * |
| Control vs. 2 Gy | **** |
| Control vs. 3 Gy | ns |
| 1 Gy vs. 2 Gy | ** |
| 1 Gy vs. 3 Gy | * |
| 2 Gy vs. 3 Gy | **** |

| Basic FGF | |
| --- | --- |
| variant | Statistical significance |
| Control vs. 1 Gy | ns |
| Control vs. 2 Gy | ns |
| Control vs. 3 Gy | * |
| 1 Gy vs. 2 Gy | ns |
| 1 Gy vs. 3 Gy | * |
| 2 Gy vs. 3 Gy | ** |

| SDF-1 alpha | |
| --- | --- |
| variant | Statistical significance |
| Control vs. 1 Gy | ns |
| Control vs. 2 Gy | ns |
| Control vs. 3 Gy | ** |
| 1 Gy vs. 2 Gy | ns |
| 1 Gy vs. 3 Gy | ns |
| 2 Gy vs. 3 Gy | ** |

| SCF | |
| --- | --- |
| variant | Statistical significance |
| Control vs. 1 Gy | ns |
| Control vs. 2 Gy | * |
| Control vs. 3 Gy | ns |
| 1 Gy vs. 2 Gy | *** |
| 1 Gy vs. 3 Gy | *** |
| 2 Gy vs. 3 Gy | **** |

| NGF-beta | |
| --- | --- |
| variant | Statistical significance |
| Control vs. 1 Gy | ns |
| Control vs. 2 Gy | ns |
| Control vs. 3 Gy | ** |
| 1 Gy vs. 2 Gy | *** |
| 1 Gy vs. 3 Gy | *** |
| 2 Gy vs. 3 Gy | **** |

| PDGF-BB | |
| --- | --- |
| variant | Statistical significance |
| Control vs. 1 Gy | ns |
| Control vs. 2 Gy | ns |
| Control vs. 3 Gy | * |
| 1 Gy vs. 2 Gy | * |
| 1 Gy vs. 3 Gy | * |
| 2 Gy vs. 3 Gy | ** |

| IFN-alpha 2 | |
| --- | --- |
| variant | Statistical significance |
| Control vs. 1 Gy | ns |
| Control vs. 2 Gy | ns |
| Control vs. 3 Gy | * |
| 1 Gy vs. 2 Gy | * |
| 1 Gy vs. 3 Gy | * |
| 2 Gy vs. 3 Gy | *** |

| IL-2R alpha | |
| --- | --- |
| variant | Statistical significance |
| Control vs. 1 Gy | ns |
| Control vs. 2 Gy | ns |
| Control vs. 3 Gy | * |
| 1 Gy vs. 2 Gy | * |
| 1 Gy vs. 3 Gy | * |
| 2 Gy vs. 3 Gy | *** |

| IL-1 alpha | |
| --- | --- |
| variant | Statistical significance |
| Control vs. 1 Gy | * |
| Control vs. 2 Gy | ns |
| Control vs. 3 Gy | **** |
| 1 Gy vs. 2 Gy | ns |
| 1 Gy vs. 3 Gy | ns |
| 2 Gy vs. 3 Gy | ns |

| TNF-beta | |
| --- | --- |
| variant | Statistical significance |
| Control vs. 1 Gy | ns |
| Control vs. 2 Gy | ** |
| Control vs. 3 Gy | * |
| 1 Gy vs. 2 Gy | ** |
| 1 Gy vs. 3 Gy | * |
| 2 Gy vs. 3 Gy | **** |

| RANTES | |
| --- | --- |
| variant | Statistical significance |
| Control vs. 1 Gy | ns |
| Control vs. 2 Gy | ns |
| Control vs. 3 Gy | ns |
| 1 Gy vs. 2 Gy | ** |
| 1 Gy vs. 3 Gy | * |
| 2 Gy vs. 3 Gy | *** |

| MIP-1 beta | |
| --- | --- |
| variant | Statistical significance |
| Control vs. 1 Gy | ns |
| Control vs. 2 Gy | ns |
| Control vs. 3 Gy | ns |
| 1 Gy vs. 2 Gy | * |
| 1 Gy vs. 3 Gy | ns |
| 2 Gy vs. 3 Gy | ns |

| CTACK | |
| --- | --- |
| variant | Statistical significance |
| Control vs. 1 Gy | ns |
| Control vs. 2 Gy | ns |
| Control vs. 3 Gy | * |
| 1 Gy vs. 2 Gy | * |
| 1 Gy vs. 3 Gy | * |
| 2 Gy vs. 3 Gy | *** |

| IL-4 | |
| --- | --- |
| variant | Statistical significance |
| Control vs. 1 Gy | ns |
| Control vs. 2 Gy | ns |
| Control vs. 3 Gy | * |
| 1 Gy vs. 2 Gy | **** |
| 1 Gy vs. 3 Gy | **** |
| 2 Gy vs. 3 Gy | **** |

| IL-7 | |
| --- | --- |
| variant | Statistical significance |
| Control vs. 1 Gy | * |
| Control vs. 2 Gy | * |
| Control vs. 3 Gy | ** |
| 1 Gy vs. 2 Gy | ns |
| 1 Gy vs. 3 Gy | ns |
| 2 Gy vs. 3 Gy | ns |

| MIG | |
| --- | --- |
| variant | Statistical significance |
| Control vs. 1 Gy | ** |
| Control vs. 2 Gy | ns |
| Control vs. 3 Gy | *** |
| 1 Gy vs. 2 Gy | * |
| 1 Gy vs. 3 Gy | ns |
| 2 Gy vs. 3 Gy | * |

| IL-9 | |
| --- | --- |
| variant | Statistical significance |
| Control vs. 1 Gy | * |
| Control vs. 2 Gy | * |
| Control vs. 3 Gy | *** |
| 1 Gy vs. 2 Gy | ** |
| 1 Gy vs. 3 Gy | ** |
| 2 Gy vs. 3 Gy | *** |

| IL-1 beta | |
| --- | --- |
| variant | Statistical significance |
| Control vs. 1 Gy | ns |
| Control vs. 2 Gy | ns |
| Control vs. 3 Gy | ns |
| 1 Gy vs. 2 Gy | * |
| 1 Gy vs. 3 Gy | ns |
| 2 Gy vs. 3 Gy | *** |

| Eotaxin | |
| --- | --- |
| variant | Statistical significance |
| Control vs. 1 Gy | ns |
| Control vs. 2 Gy | * |
| Control vs. 3 Gy | * |
| 1 Gy vs. 2 Gy | ** |
| 1 Gy vs. 3 Gy | ns |
| 2 Gy vs. 3 Gy | **** |

| GM-CSF | |
| --- | --- |
| variant | Statistical significance |
| Control vs. 1 Gy | ns |
| Control vs. 2 Gy | ns |
| Control vs. 3 Gy | * |
| 1 Gy vs. 2 Gy | ** |
| 1 Gy vs. 3 Gy | ** |
| 2 Gy vs. 3 Gy | *** |

| IL-2 | |
| --- | --- |
| variant | Statistical significance |
| Control vs. 1 Gy | ns |
| Control vs. 2 Gy | ns |
| Control vs. 3 Gy | ** |
| 1 Gy vs. 2 Gy | ns |
| 1 Gy vs. 3 Gy | ns |
| 2 Gy vs. 3 Gy | * |

| IL-13 | |
| --- | --- |
| variant | Statistical significance |
| Control vs. 1 Gy | ns |
| Control vs. 2 Gy | ns |
| Control vs. 3 Gy | * |
| 1 Gy vs. 2 Gy | ns |
| 1 Gy vs. 3 Gy | ns |
| 2 Gy vs. 3 Gy | ns |

| IL-17 | |
| --- | --- |
| variant | Statistical significance |
| Control vs. 1 Gy | ns |
| Control vs. 2 Gy | ns |
| Control vs. 3 Gy | * |
| 1 Gy vs. 2 Gy | ns |
| 1 Gy vs. 3 Gy | ns |
| 2 Gy vs. 3 Gy | ns |

| IL-3 | |
| --- | --- |
| variant | Statistical significance |
| Control vs. 1 Gy | ns |
| Control vs. 2 Gy | ns |
| Control vs. 3 Gy | ns |
| 1 Gy vs. 2 Gy | ns |
| 1 Gy vs. 3 Gy | ns |
| 2 Gy vs. 3 Gy | ns |

| M-CSF | |
| --- | --- |
| variant | Statistical significance |
| Control vs. 1 Gy | ns |
| Control vs. 2 Gy | * |
| Control vs. 3 Gy | ns |
| 1 Gy vs. 2 Gy | ** |
| 1 Gy vs. 3 Gy | ** |
| 2 Gy vs. 3 Gy | *** |

| MIP-1 alpha | |
| --- | --- |
| variant | Statistical significance |
| Control vs. 1 Gy | ns |
| Control vs. 2 Gy | ns |
| Control vs. 3 Gy | ns |
| 1 Gy vs. 2 Gy | * |
| 1 Gy vs. 3 Gy | ns |
| 2 Gy vs. 3 Gy | * |
